# Genomic Catastrophe Defines the Evolutionary Trajectory of Adrenocortical Carcinoma

**DOI:** 10.1007/s12022-026-09922-2

**Published:** 2026-06-10

**Authors:** Samuel Backman, Fredrik Axling, Liang Zhang, Johan Botling, C Christofer Juhlin, Elham Barazeghi, Britt Skogseid, Branislav Klimácek, Matilda Annebäck, Jan Zedenius, Olov Norlén, Peter Stålberg, Joakim Crona, Tobias Åkerström

**Affiliations:** 1 Department of Surgical Sciences, Uppsala University Hospital, 75185 Uppsala, Sweden; 2 Department of Medical Sciences, Uppsala University, Uppsala, Sweden; 3 Department of Laboratory Medicine, Institute of Biomedicine, University of Gothenburg, Gothenburg, Sweden; 4 Department of Oncology - Pathology, Karolinska Institutet, Stockholm, Sweden; 5 Department of Clinical Pathology and Cancer Diagnostics, Karolinska University Hospital, Stockholm, Sweden; 6 Department of Molecular Medicine and Surgery, Karolinska Institutet, Stockholm, Sweden; 7 Department of Breast, Endocrine Tumors and Sarcoma, Karolinska University Hospital, Stockholm, Sweden

**Keywords:** Adrenocortical carcinoma, Tumour evolution, Copy number alterations, Whole genome sequencing, Endocrine cancer

## Abstract

The mechanisms underlying how adrenocortical carcinoma (ACC) progresses into a metastatic and lethal disease remain poorly understood. To address this, we performed comprehensive genomic analyses to delineate the evolutionary trajectory of advanced ACC. Fresh frozen tumour samples (*n* = 29) were obtained from nine patients, all of whom had matched primary and relapse specimens, including recurrent (*n* = 4) and metastatic (*n* = 11) lesions. In four patients, multiple primary and metastatic samples were available, enabling detailed evolutionary comparisons. All tumours underwent whole-genome sequencing, RNA sequencing, and DNA methylation profiling. Our analyses revealed that seven of nine patients exhibited global loss of heterozygosity (LOH) often followed by whole-genome doubling, resulting in copy-neutral LOH. SNP-based analyses indicated that these alterations occurred as a single catastrophic event that was conserved across all matched samples within each patient, suggesting that this event constitutes a truncal feature of the evolutionary tree. These results support a model in which chromosomal aneuploidy is important in ACC tumourigenesis, potentially distinguishing carcinomas from benign adrenal adenomas. This may explain the rarity of adenoma-to-carcinoma transformation and suggest a diagnostic and therapeutic relevance of chromosomal instability in ACC.

**Supplementary Information:**

The online version contains supplementary material available at 10.1007/s12022-026-09922-2.

## Introduction

Adrenocortical carcinoma (ACC) is an ultra-rare disease with variable prognosis, that is particularly poor in the metastatic setting [1, 2]. Surgery is considered the only curative treatment and is recommended for patients amenable to complete resection [3]. Patients with confirmed or suspected ACC should be evaluated for tumour resection without delay, as clinical experience shows that these tumours can progress rapidly, resulting in a shift of the therapeutic window from a curative to a palliative setting. The underlying biological rationale for this behaviour is unknown. Whether ACC originate from cells that have intrinsic metastatic capacity, or if they acquire metastasis-initiating events is poorly investigated [4].

While adrenal cortical adenomas are characterized by a low mutation burden and few chromosomal aberrations, ACC harbour mutations in established cancer driver genes and/or undergo large copy number alterations (CNA). Specifically, mutations in genes associated with TP53/RB, WNT/Beta-catenin and/or chromatin signalling pathways, as well as chromosomal instability have been proposed as driving mechanisms in tumorigenesis [5, 6]. The Cancer Genome Atlas (TCGA) further highlighted the importance of large-scale CNA in ACC, emphasizing the roles of both loss of heterozygosity (LOH) and whole-genome doubling (WGD) [6]. How these aberrations evolve during ACC tumorigenesis remains unknown.

The understanding of metastatic ACC is limited by the poor availability of biomaterials, especially paired primary and relapse samples with adequate tissue quality for multi-omics analyses. Previous studies using pan-genomic sequencing on metastatic ACC have been conducted without having matched primary tumours as comparison. However, the molecular landscapes observed in these studies on ACC metastases appear to be very similar to that of primary ACC [7–9]. Interestingly, a complex pattern of intratumoural heterogeneity has been shown in other studies using targeted sequencing techniques [4, 10, 11]. Mutations in driver genes were shown to occur heterogeneously between samples, the clinical relevance of which is unknown, and these findings did not clarify the mechanism driving ACC tumour formation or the events that contribute to metastatic seeding.

Our hypothesis in the current study is that some ACC acquire metastasis-initiating events, while others have intrinsic metastatic capacity and are therefore “born bad”, carrying early acquired large CNA. To address these questions, we conducted a multi-omics characterization of paired frozen samples from nine patients with matched primary and metastatic/recurrent ACCs and applied evolutionary analyses.

## Materials and Methods

### Patient Selection

We evaluated all patients with histologically proven ACCs available in the Endocrine surgery biobank at Uppsala University hospital. In one patient, two samples obtained through Karolinska University Hospital were also included. Inclusion and exclusion criteria are shown in Supplementary Fig. 1A. Following ethical approval (Etikprövningsnämnden i Uppsala Dnr 2012/422, and Stockholm Dnrs 2001 − 136 and 2020/04226) and informed consent, tumour and normal tissue samples obtained during surgery had all been flash frozen and stored at -70 °C. DNA and RNA extraction was performed using the AllPrep DNA/RNA/miRNA Universal Kit, (Qiagen, Hilden, Germany). Prior to inclusion in the study, hematoxylin & eosin-stained tissue sections were scrutinized by an expert endocrine pathologist to ensure adequate tumour cell content of > 50% and a correct diagnosis of ACC. In four cases an endocrine pathologist reevaluated the Weiss criteria due to missing data in the clinical patient charts. Ki-67 expression was acquired from the clinical data inventory, or when not available, Ki-67 staining was performed using the antibody HPA068322, dilution 1:100, Atlas Antibodies.

### Sequencing and Array Analyses

Whole genome sequencing libraries were prepared from 1 µg of DNA using the TruSeq PCRfree DNA sample preparation kit. Paired-end sequencing with a 150 bp read length was performed on the NovaSeq 6000 system, SP flowcell and v1 sequencing chemistry. RNA sequencing libraries were prepared from 1 µg total RNA using the TruSeq stranded total RNA library preparation kit with RiboZero Gold treatment and the libraries were sequenced using NovaSeq S4 flowcell, paired-end 150 bp read length, and v1 sequencing chemistry. The sequencing was performed at the Uppsala node of SciLife Laboratory. Methylation profiling was performed using the Illumina Infinium EPIC array (v1), after bisulfite treatment using the EZ DNA MethylationTM Kit (Zymo research). The sequencing and array analyses were performed at the Uppsala node of SciLife Laboratory.

### Bioinformatics Analyses

Processing of the raw whole genome sequencing and DNA methylation data was carried out as previously described [12]. Briefly, the whole genome sequencing data was analysed using a lightly modified version of the nf-core/Sarek v2.6 pipeline. Copy number calling was performed using Sclust, with manual recalibration of ploidy as necessary. The Sclust mutation copy number estimates were used to estimate the fraction of mutations occurring prior to WGD / copy number neutral LOH (cnLOH) by dividing the number of mutations in cnLOH segments with estimated mutation copy number of 2 by the number of mutations in cnLOH segments with estimated copy number 1 or 2. Subclonal reconstruction was performed with PyClone-VI as previously described [12], with the exception that all clusters were included in visualizations. Mutation signature assignment was carried out using SigProfilerAssignment with COSMIC v3.4 signatures. RNA sequenced reads were quality trimmed by a Phred > 30 score threshold and adapter sequence removal by Trim Galore [13]. Transcript abundance estimates were derived via Salmon [14], incorporating 100 bootstrap replicates to account for technical variability as well as bias correction for GC content and sequence composition, against the GRCh38.108 transcriptome with included genome decoys [15]. After quantification, transcript-level data were imported into DESeq2, where they were summarized at the gene level with BioMart [16] and Tximeta [17] and used for downstream differential expression analysis. Differentially expressed genes were subsequently investigated for interaction networks by the STRING database [18], while network structures were visualized in Cytoscape [19]. Enrichment for biological pathways and gene ontology terms among the differentially expressed genes was performed using g: Profiler [20]. Code to reproduce key findings and figures has been deposited in a publicly available repository (https://github.com/sabackman/ACC2025). Coefficient of variation was calculated using (Standard deviation of mutations/mean of mutations). Focal copy number alteration was defined using empirically determined thresholds as follows: gain, copy number ≥ ploidy + 2; high-level amplification, copy number ≥ ploidy + 3; homozygous deletion, copy number = 0. For structural variants, in addition to Manta SV, calls generated using the Sarek pipeline, somatic SVs were called with Delly [21] using the standard settings. The output from both callers was standardized using OctopuSV correct [22], and finally a high-confidence call set was generated by identifying calls made by both callers using OctopuSV merge with the “-- intersect” parameter.

## Results

**Fig. 1:**
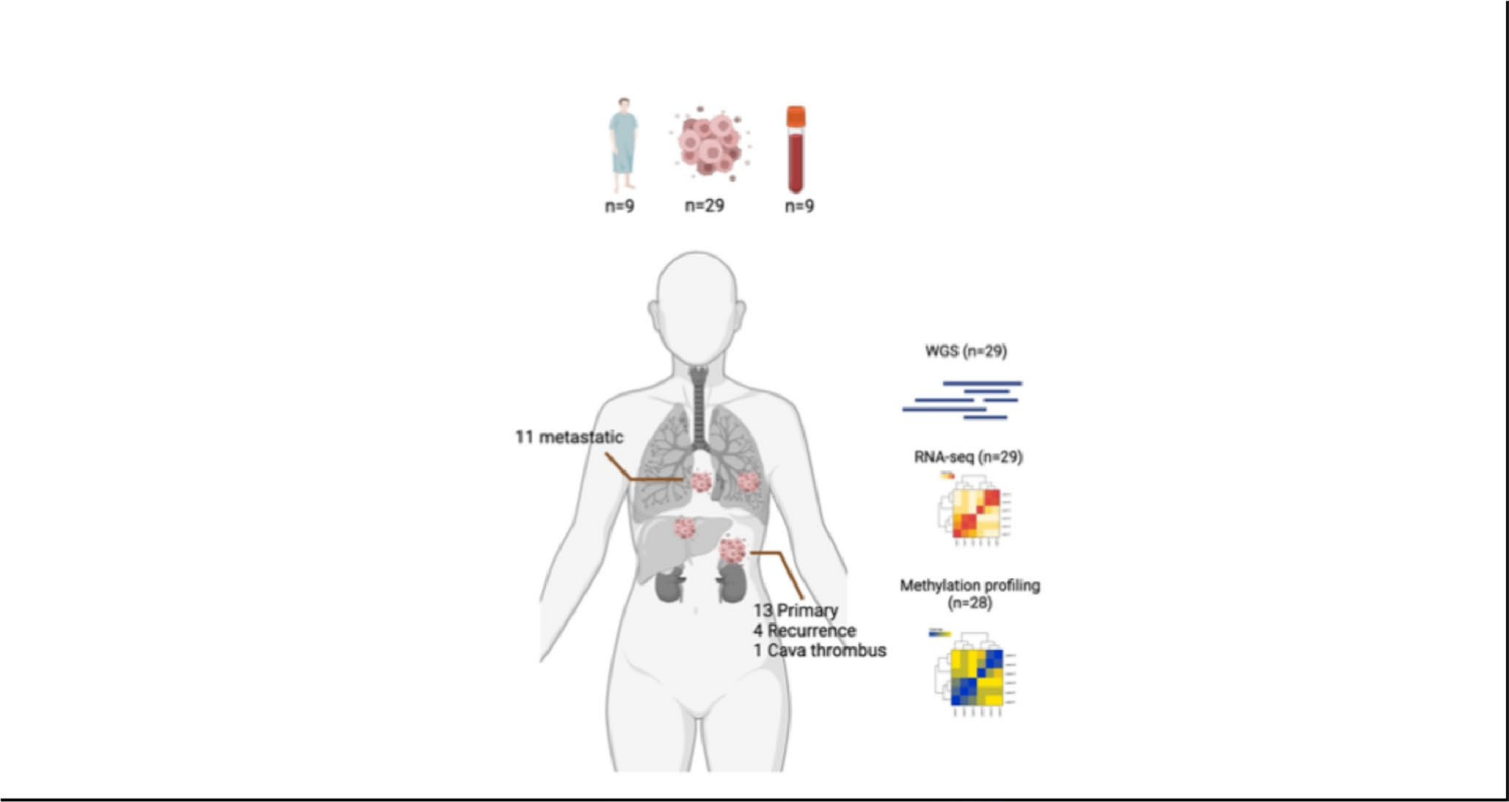
Overview of the different genetic analyses and included specimens in the cohort

To define the genomic architecture and evolutionary trajectories of advanced and sporadic ACC, we interrogated 68 patients and identified nine with paired frozen primary and relapse samples and matched normal tissue. Twenty-five unique tumour samples underwent multi-omics profiling. In four patients, two spatially distinct regions from the primary tumour were analysed, resulting in a total cohort of 29 samples (Fig. 1).

The cohort was clinically aggressive: at the time of writing all but three patients had died of their ACC (Supplementary Tables 1–2).

### Single Nucleotide Variant/Small Indel Detection

**Fig. 2:**
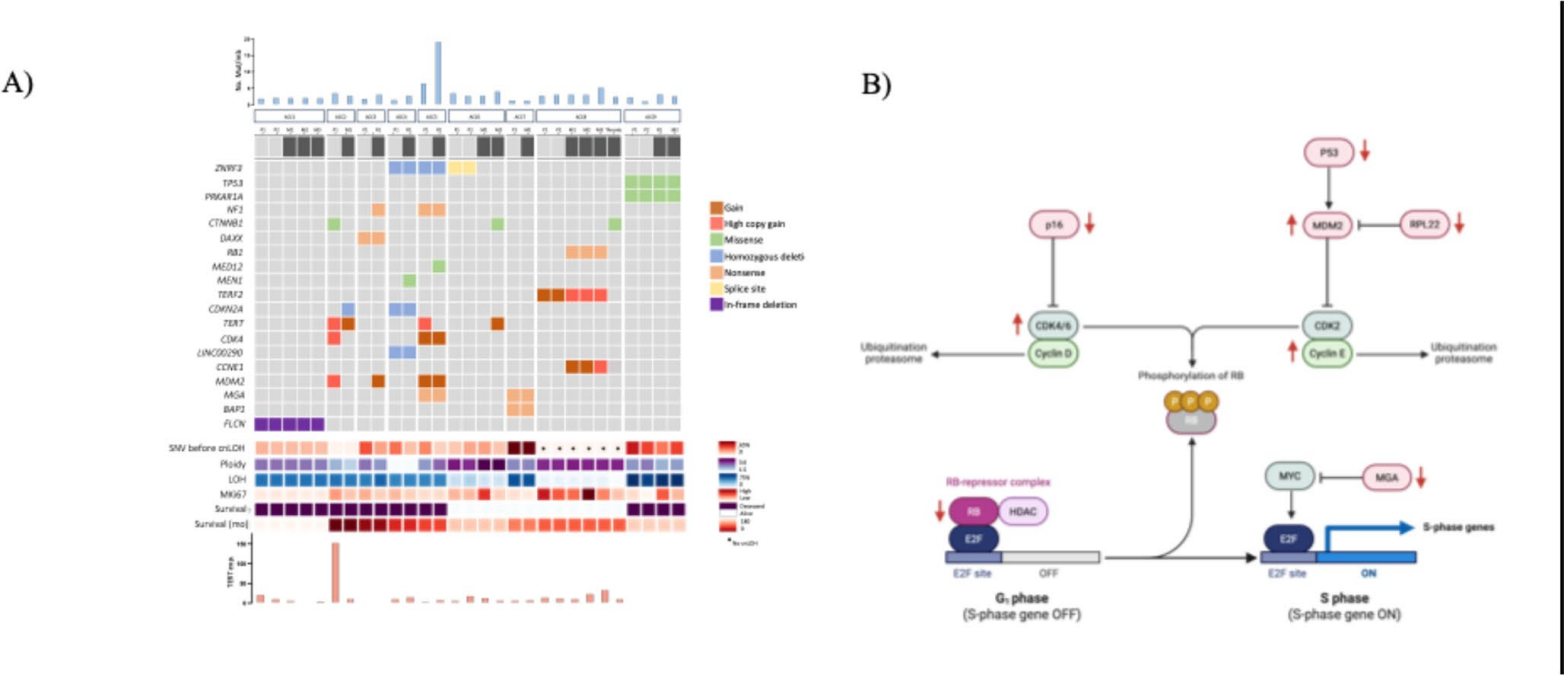
(**A**) Oncoprint summarizing somatic mutations and copy number alterations identified in matched primary (P), metastatic (M), and recurrent (R) adrenocortical carcinoma (ACC) samples. Each column represents a tumour sample. TMB: Tumour mutation burden. SNV: Single nucleotide variants. LOH: Loss of heterozygosity in percent. MKi67, TERT: normalised expression using RNA sequencing. (**B**) Summary of ACC driver genes affecting the G1-S phase. P16 (*CDKN2A*), Cyclin E (*CCNE1*)

The median tumour mutation burden (TMB) was 3.18 mutations per megabase. There was a higher number of mutations in metastatic compared with primary tumour samples, the difference was not statistically significant (median 4.44 vs. 2.59, *p* = 0.44). Although, the overall patterns of single nucleotide variants (SNV) mirrored previous ACC datasets, pronounced spatial heterogeneity was observed, with 41% of known ACC drivers occurring in a non-uniform manner within individual patients. Early clonal SNV alterations were restricted to *TP53*, *PRKAR1A*, and *DAXX*, whereas *RB1*, *MED12*, *NF1*, and *CTNNB1* were subclonal (Fig. 2, Supplementary Table 3).

Focusing on variants with high allele frequency, we identified four additional putative early drivers: *FLCN*, *MGA*, *BAP1*, and *RNF43*, all occurring at high variant allele frequency and with known tumour-suppressive roles in other cancer types. Interrogation of external ACC cohorts, using cbioportal [23–25], confirmed recurrent mutations in these genes (1.9–5.5%).

Mutational signature analyses showed predominant enrichment of the pan-cancer SBS5 and SBS40 signature (Supplementary Fig. 2). The two tumours from ACC5 displayed the Microsatellite instability-associated SBS44 signature. These samples also had the highest number of somatic mutations (TMB 6.5 and 19.3). In the germline data we noted a previously unrecognized frameshift mutation in *MSH6* (p.Met799fs). This patient was later diagnosed with colon carcinoma, at which point the diagnosis of Lynch syndrome was made by clinical genetics. Additionally, in ACC8, one tumour was exposed to cisplatin and acquired a SBS31 platinum-associated mutation signature.

### Copy Number Analysis

**Fig. 3:**
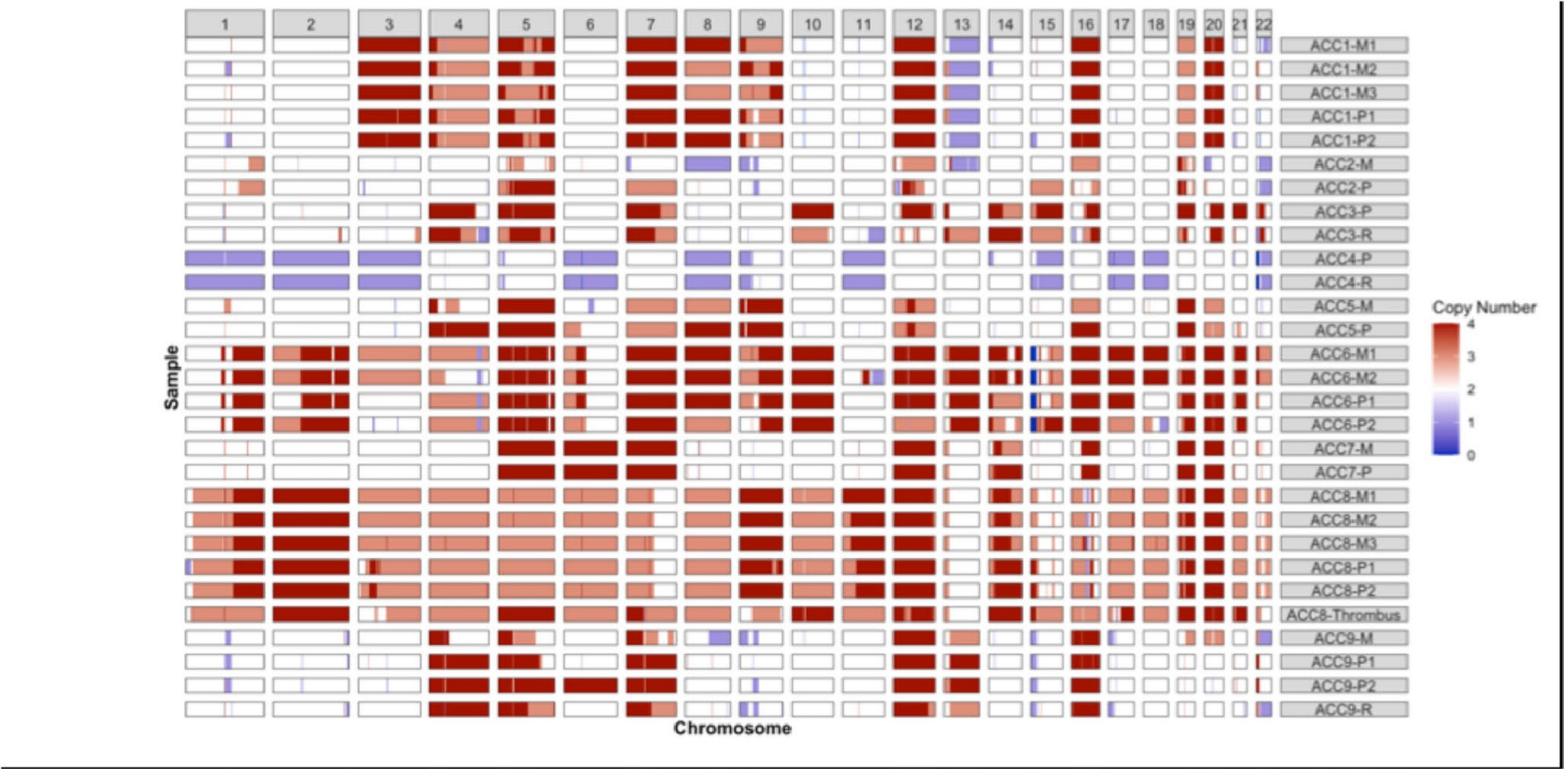
Copy number alterations of the cohort. Heatmap showing copy number profiles for all analysed ACC samples, including primary (P), metastatic (M), and recurrent (R) tumours. Each column represents a chromosome (1–22), and each row corresponds to an individual sample

Adrenocortical carcinomas display a unique copy-number pattern across their genomes [26], with frequent chromosomal LOH and subsequent WGD [5–7, 9]. We confirmed this strikingly conserved copy-number phenotype. Seven of nine patients exhibited global LOH, followed by WGD in six cases, consistent with the canonical “chromosomal” ACC subtype [6]. CNA patterns were remarkably stable across tumour sites, indicating that chromosomal catastrophe occurs early and is clonally propagated (Fig. 3, Supplementary Fig. 3).

**Fig. 4:**
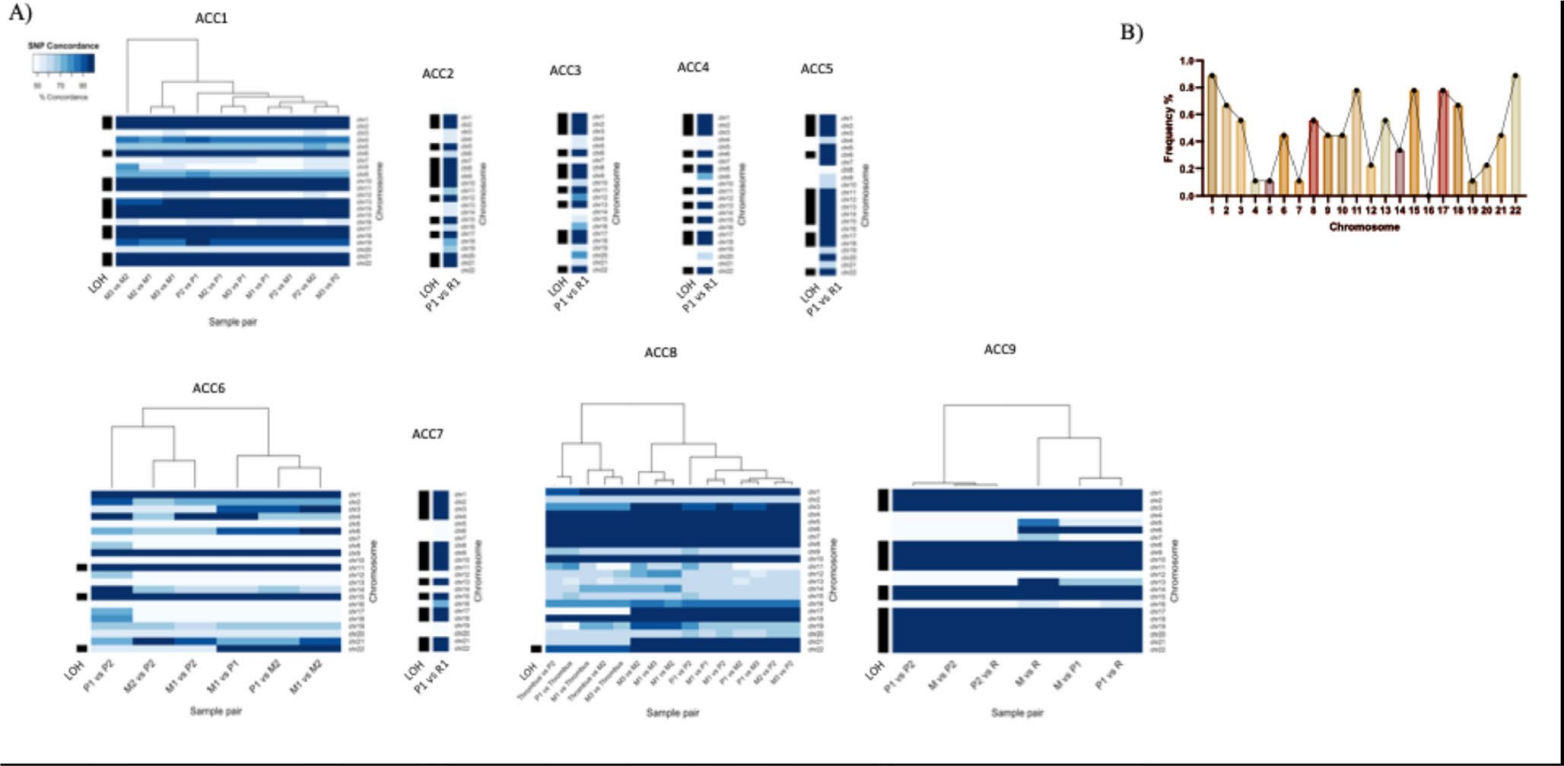
(**A**) Heatmap showing SNP concordance between paired primary and relapse tumour samples across chromosomes. The colour gradient represents the percentage of SNP concordance between samples. Black boxes on the left mark chromosomes showing loss of heterozygosity (LOH). (**B**) Frequency of LOH across chromosomes in the cohort. Each bar represents the proportion of samples showing LOH for the corresponding chromosome

SNV timing analyses confirmed this model: on average, 76% of mutations accumulated after cnLOH (Supplementary Table 4). Furthermore, by comparing concordant/discordant SNPs among tumour pairs, a striking overlap in the retained allele was observed in LOH positive patients (Fig. 4A).

Together these results suggest a single truncal catastrophic event, leading to large scale losses of the genome, followed by reconstitution of a diploid state through WGD. The early establishment of chromosomal CNAs, was exemplified in two patients ACC2 (cnLOH positive) and ACC8 (cnLOH negative), both these patients had extensive CNAs across their genomes. Remarkably, in ACC2, only 0.5% of mutations occurred prior to cnLOH. Likewise, in ACC8, a shared CNA profile was observed across samples, however sequencing of a tumour thrombus showed that only 2% of mutations occurred in the most recent common ancestor. These examples place large CNA at the root of the evolutionary tree.

Importantly, for cnLOH samples, regions affected by these events were recurrent among tumours, suggesting a selection for this signal (Fig. 4B, Supplementary Table 5) [26], with chromosomal LOH in > 50% of patients on chromosomes 1, 2, 3, 8, 11, 13, 15, 17, 18, and 22, while often sparing chromosomes 4, 5, 7, 12, 16, 19, and 20. Similar LOH patterns have been reported in chromophobe renal cell carcinoma, pancreatic neuroendocrine tumours and oncocytic thyroid carcinoma [27–31].

To find alterations linked to this CNA signal, we examined mutations shared across tumour sites together with clonal SNVs. We noted shared mutations in *TP53*, the canonical facilitator of WGD, in all tumours of ACC9, appearing at an early stage in the disease evolution [32]. A clonal *BAP1* mutation in ACC7 was observed, previously implicated in spindle-assembly checkpoint (SAC) dysfunction and aneuploidy tolerance [32]. We also noted amplification of *MDM2* in three patients, causing bypass of *TP53* induced senescence [33], and previously linked to WGD positive ACC [6]. Additional heterogeneous alterations in *CCNE1*, *CDK4*, *CDKN2A*, *ATR*, and *RB1*, were identified in six of nine patients, consistent with an importance of deregulated G1–S transition in aneuploid ACC tumours (Fig. 2B).

Cancers with high chromosomal instability may develop a dependency on the SAC, which enables cells to complete mitosis despite extensive aneuploidy and protects them from apoptosis [34]. As most ACC display chromosomal instability, we speculated that they may become dependent on a functional SAC to maintain tumour viability. Supporting this, high expression of the SAC regulator *BUB1B* has been identified as a strong predictor of poor outcome in ACC [35]. To explore whether this dependency extends beyond *BUB1B*, we analysed SAC-associated genes in the TCGA-ACC cohort using GEPIA [36]. Several SAC and mitotic regulators, including *KIF11*,* ZWINT*,* AURKA*, and *KIF18A*, emerged among the most prognostic genes in ACC, outperforming many canonical cancer drivers. Expanding this analysis to broader SAC components revealed a consistent pattern: high expression of SAC machinery correlated with inferior overall survival (Supplementary Fig. 4).

### Structural Rearrangements

To further investigate genomic alterations, we analysed structural rearrangements. In line with the small mutation data, there was no difference between primary and recurrent/metastatic samples with regard to structural variant load (118.8 vs. 178.6, *p* = 0.2 by the Wilcoxon rank sum test with normal approximation, Supplementary Fig. 5). In some patients there was a substantial overlap between samples, suggesting early clonal alterations. Furthermore, in four patients these localized regions were associated with hypermutation, *kataegis* (Supplementary Fig. 5).

### Tumour Evolution

Multi-sample analysis revealed a branched evolutionary pattern in all patients, with marked heterogeneity in shared and private mutation burdens between primary and metastatic lesions (median coefficient of variation 0.5), consistent with early divergence and parallel evolution (Supplementary Table 6). PyClone-VI subclonal inference demonstrated marked shifts in clonal composition over time, with most relapses exhibiting dominant clonal sweeps (Supplementary Fig. 6). These findings highlight pronounced intrapatient heterogeneity and dynamic clonal restructuring of SNV throughout ACC progression, in stark contrast to the relatively stable CNA profiles.

### Genetic investigations of longitudinal samples

**Fig. 5:**
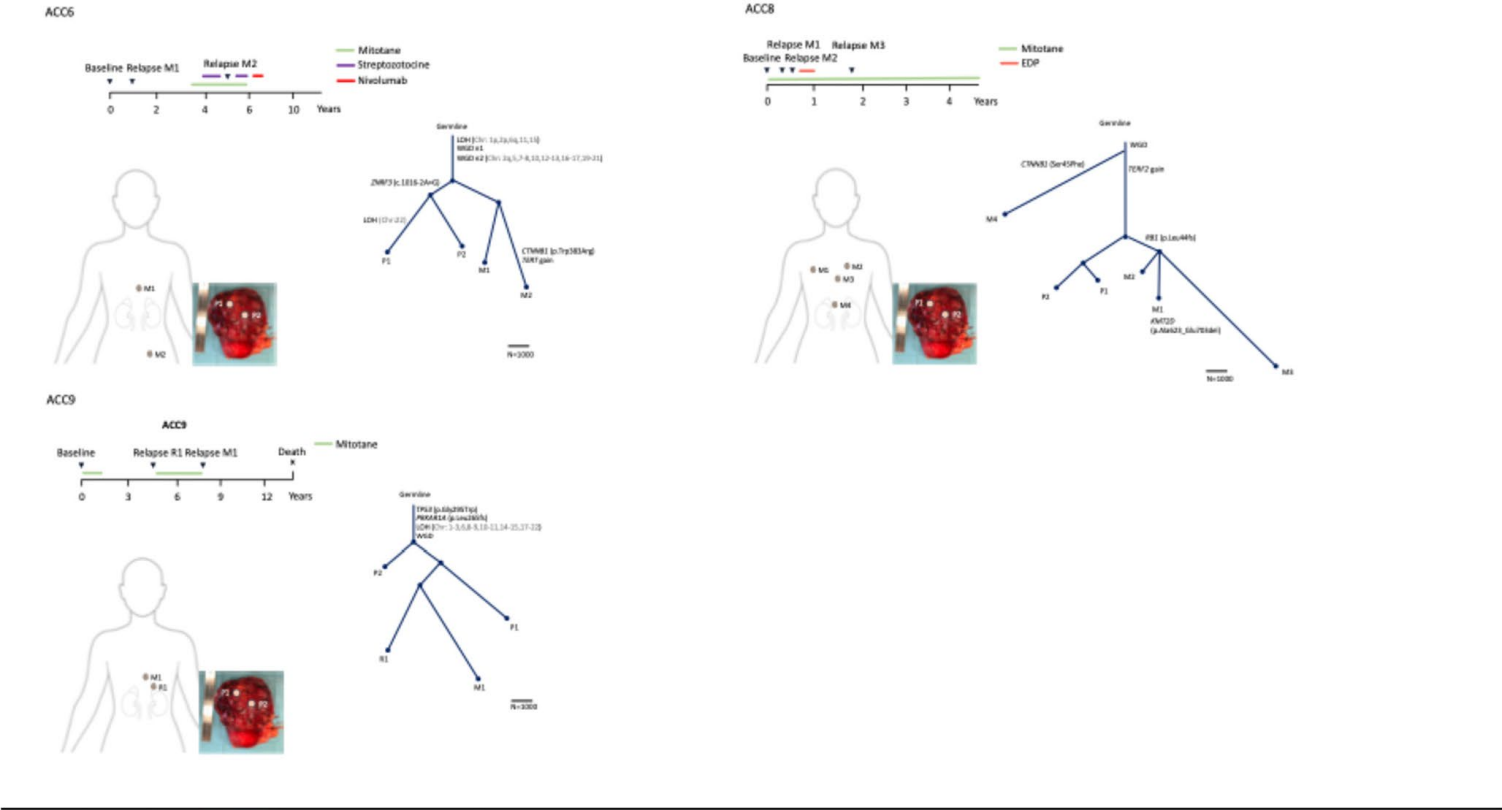
Depiction of three patients with multiple samples in the cohort. The phylogenetic tree was created based on unique and shared mutations among samples using the raw phylogenetic trees in Supplementary Fig. 8. P: Primary, R: Recurrence, M: Metastasis, ACC: Adrenocortical carcinoma, WGD: Whole genome doubling, EDP: Etoposide, doxorubicin, cisplatin

To further investigate tumour evolution, we analysed longitudinal samples from three patients (ACC6, ACC8, and ACC9; Fig. 5).

Patient ACC6 had undergone a computed tomography of her abdomen four years prior to diagnosis, showing a 10 × 7 mm tumour, with an attenuation of 33 Hounsfield units (Supplementary Fig. 7). Four years later, she was diagnosed with a 10 cm ACC. The primary tumour showed 9/9 Weiss criteria and a Ki-67 of 25%, the patient later developed lung, lymph-node, and an ovarian metastasis (Ki-67 49%). After resection of the ovarian mass, the remaining lung and lymph-node metastases responded remarkably to nivolumab and she remains in complete response four years after her last surgery. A truncating splice-site mutation in *ZNRF3* (c.1016–2 A > G) was heterogeneously present in the primary tumour but absent from metastases, indicating early branching and limited metastatic relevance (Supplementary Fig. 8). Convergent WNT-pathway activation likely occurred through *CTNNB1* (p.Trp383Arg) in the ovarian metastasis and *RSPO2* (p.Ser103Phe) in the lymph-node metastasis. A striking overlap of CNA was observed between sample sites (Supplementary Fig. 3). 16% of SNVs preceded genome-wide LOH and WGD, confirming early chromosomal aneuploidy followed by later WNT-pathway mutations.

In ACC8, a tumour thrombus that was resected together with the primary tumour carried a *CTNNB1* gain-of-function mutation (p.Ser45Pro) not found elsewhere. Only 2% of SNVs were shared with other samples, indicating extremely early divergence. All samples, including the thrombus, shared a similar aneuploid genome, confirming that CNA events occurred early. A *TERF2* gain was detected in all samples except the thrombus, while metastases shared a *CCNE1* gain and *RB1* loss-of-function mutation. Extensive SNV heterogeneity and clonal sweeps were observed in metastases.

In ACC9, two primary-tumour regions, a recurrence, and a metastasis were sequenced. Interestingly, the primary tumour was only 3.5 cm, displayed a Ki67 index of less than 1%, and a Weiss score of three points. She later developed liver and pancreatic metastases and ultimately died from her disease. Large chromosomal aberrations occurred in both the primary and relapse samples. In addition, a truncating *PRKAR1A* and missense *TP53* mutations were clonal across all samples and occurred prior to cnLOH.

### Transcriptional Patterns and Methylation analysis

RNA-sequencing and methylation analyses showed that tumour samples clustered by patient rather than by tumour site (Supplementary Figs. 9–10). We detected no evidence of steroidogenic dedifferentiation between primary and relapse samples, nor any effect of mitotane exposure (Supplementary Table 7). Relapse tumours exhibited enriched dysregulation of genes involved in chromatin organisation, nucleosome structure, and DNA packaging, with a prominent signal in histone-modifying pathways (Supplementary Fig. 9B-C). Integration with TCGA-ACC data revealed that 138 of relapse associated genes were significantly correlated with patient survival (Supplementary Tables 8–10).

## Discussion

The genomic landscape of ACC has been defined in recent years through large sequencing efforts, establishing recurrent driver-alterations in WNT signalling, TP53/RB pathways, and chromatin regulators [5, 6, 37]. Yet, because most tumours analysed to date were primary lesions, the evolutionary dynamics underpinning metastatic progression have remained unresolved. Analyses of paired primary and metastatic samples have hinted at extensive molecular heterogeneity [6, 10, 11], but sample scarcity has limited deeper evolutionary inference.

Using a unique cohort of fresh-frozen, paired primary and relapse samples, we delineate the longitudinal evolution of advanced ACC at whole-genome resolution. Transcriptomic profiling showed an association with chromatin-associated, nucleosomal and DNA-packaging genes, programmes linked to open-chromatin states, and epithelial to mesenchymal transitions [38, 39]. Given that many canonical ACC drivers encode chromatin or histone regulators, this points to chromatin deregulation as a vulnerability in advanced disease [5, 6].

We observed heterogeneity in known driver alterations, including *ZNRF3* and *CTNNB1*, supporting a model in which WNT activation can be achieved through multiple, non-redundant genomic routes [6, 10, 11]. Conversely, some mutations, including *TP53*, were fully clonal, consistent with their acquisition early in tumourigenesis. Several putative early drivers (*RNF43* [40, 41], *BAP1* [42], *MGA* [43], *FLCN* [44, 45]) were identified through mutational-timing analyses. These genes converge on pathways regulating WNT signalling, mitotic fidelity, MYC-driven proliferation, and mTOR activity.

In ACC5, a microsatellite instability mutational signature was observed. No somatic explanation for this signal was identified, but a germline nonsense mutation in *MSH6* was discovered. Although the patient initially presented with apparently sporadic ACC, the subsequent development of colorectal carcinoma led to a diagnosis of Lynch syndrome. Screening for germline mutations in apparently sporadic ACC have shown *MSH* gene mutations in approximately 3% [6, 46–49], highlighting the importance of considering germline events in ACCs.

Using the high resolution of WGS we analysed genomic rearrangements, and observed a high degree of overlap in alterations between primary and relapse samples, suggesting relatively early events. Areas affected by these rearrangements displayed signs of regional hypermutation signatures (kataegis) in some of our patients, a phenomenon previously described in pediatric adrenocortical tumours [50].

A key insight from this study is the dominance of large-scale chromosomal events and its involvement in ACC evolution. In the study by Zheng et al. the importance of large structural variations in ACC was highlighted. They described that LOH positive tumours likely were precursors to WGD positive tumours. Furthermore, they also described that WGD tumours displayed upregulation of telomere pathways, cell cycle regulation and DNA replication repair [6]. Nearly all our tumours underwent widespread LOH followed by WGD, with ~ 75% of SNVs arising after these events, placing chromosomal catastrophe at the trunk of the evolutionary tree. The LOH pattern was highly stereotyped, sparing chromosomes enriched for essential genes, including chromosome 5, where *TERT* resides, perhaps reflecting an important mechanism to protect against telomere attrition [51]. Interestingly, in pancreatic neuroendocrine tumours, also defined by large-scale LOH, most display an alternative lengthening of telomeres (ALT) phenotype [28].

Selection for large-scale LOH involves increased acquisition of damaging mutations in tumour suppressor genes. However, essential genes are likewise vulnerable to loss, leading to negative selection of these clones [52]. Subsequent WGD creates cnLOH and a survival advantage for clones carrying these alterations. Interestingly, in pan-cancer analyses, ACC exhibits a unique CNA profile characterized by extensive LOH [26] followed by WGD [32]. In the TCGA database the rate of genome-wide LOH vary between tumour types reaching 45% in ACC and only 0.01% in thyroid carcinomas [26]. The most common genetic association with WGD is *TP53* mutations, observed in nearly half of tumours [32]. In *TP53* wild type, WGD positive tumours, other associated alterations include amplification of *MDM2*, *CCNE1*, *CCND1* and *CDK4*, and loss of function mutations in *BAP1*,* CDKN2A* and *RB1*. Across cancers, 32% of TP53–wild-type tumours with WGD harbour defects in the E2F/G1 regulatory axis [32], a pathway critically required for tolerating tetraploidy and permitting continued cell-cycle progression. It is striking that many of these alterations, are recurrent in ACC, supporting a model in which deregulated E2F/G1 and G1-S transition facilitates survival after catastrophic chromosome alterations in ACC. Most of the alterations in this pathway were heterogeneous across tumour regions, suggesting that although E2F/G1 dysregulation is advantageous, it is not always the initiating event, but may rather be a later adaptation for aneuploid clones.

Previous analyses of aneuploid tumours have shown an increased dependence on mitotic regulators [34]. Genes involved in the spindle assembly checkpoint (SAC) notably *BUB1B*, *AURKA*, *KIF11*, *ZWINT* and *KIF18A* were among the strongest survival predictors in the TCGA cohort. These findings may suggest that aneuploid ACC cells become reliant on a working mitotic machinery to avoid mitotic catastrophe [34]. This dependency is therapeutically attractive, as SAC inhibitors induce apoptosis in ACC cells [53–55] and several agents are already in clinical development in advanced carcinomas [56, 57].

The early emergence and clonal conservation of large-scale CNAs have important implications for tumour origin and clinical management of ACCs. Adrenocortical adenomas rarely harbour widespread CNA [58–60], and only about 10% of ACC have quiet genomes. Moreover, adenoma-to-carcinoma transitions are exceedingly rare in ACC [61, 62]. Furthermore, except for *CTNNB1* mutations, which occur clonally in adenomas and often subclonal in malignant tumours [60, 63], no other ACC driver mutations are overlapping [59]. Retrospective imaging studies show that when prior scans exist, ACCs frequently appear as small, radiographically non-benign lesions that progress rapidly, supporting a “born bad” model [64, 65]. This was highlighted in ACC6, where a small 10 mm nodule was seen only four years prior to the diagnosis of a 10 cm ACC. This tumour harboured widespread CNA with no apparent clonal SNV driver. However, exceptions do occur and some apparently benign lesion may develop into ACC after a very long period without growth [62]. Genotyping these lesions would likely give more information on their specific evolution.

No recurrent genomic metastatic switch was identified in this cohort. Hence, determining a clear size indication for surgery may be challenging. This is in line with previous sequencing on metastatic ACC showing no clear difference in metastatic tumours [7, 9]. The distinct CNA profile of ACC compared with adenomas could potentially be utilised in patients with tumours of unknown malignant potential, as exemplified by ACC1 and ACC9, in which the primary tumours had low proliferation and low Weiss scores, but displayed the typical cnLOH signal. Furthermore, preoperative liquid biopsies with CNA analysis in patients with suspicious adrenal tumours could also be of interest. Additionally, our result also suggests that determining prognosis using SNVs should be approached with caution, and that CNA and methylation profiles may offer better alternatives.

Finally, beyond potential treatment targets in the mitotic machinery, the pervasive LOH landscape of ACC may create an opportunity for allele-specific therapeutic strategies. Essential genes expressed from a single retained allele may be selectively targetable using allele specific CRISPR targeting. Also, heterozygous loss of function mutations in proteins may cause selective damage by therapeutic compounds through the loss of expression of wild type alleles in tumour tissues [66, 67], or by the use of chimeric antigen receptor T-cells (CAR-T) [68]. Given that ACC exhibits one of the most extreme LOH profiles of any human cancer, such approaches warrant further investigation.

### Limitations

The cohort used in this investigation only included advanced ACC. Due to the small number of patients the applicability to all ACC tumours is limited. Furthermore, as all patients underwent surgery for their relapse, a selection of patients with a less aggressive disease process may have occurred. To mitigate these issues, we verified many of our findings using other published reports on ACC. Moreover, as ACC represents a rare disease and removal and proper storage of high-quality fresh frozen metastatic tissue is even rarer, we believe that this report adds to the current knowledge of these tumours, despite its inherent biases.

## Conclusions

**Fig. 6:**
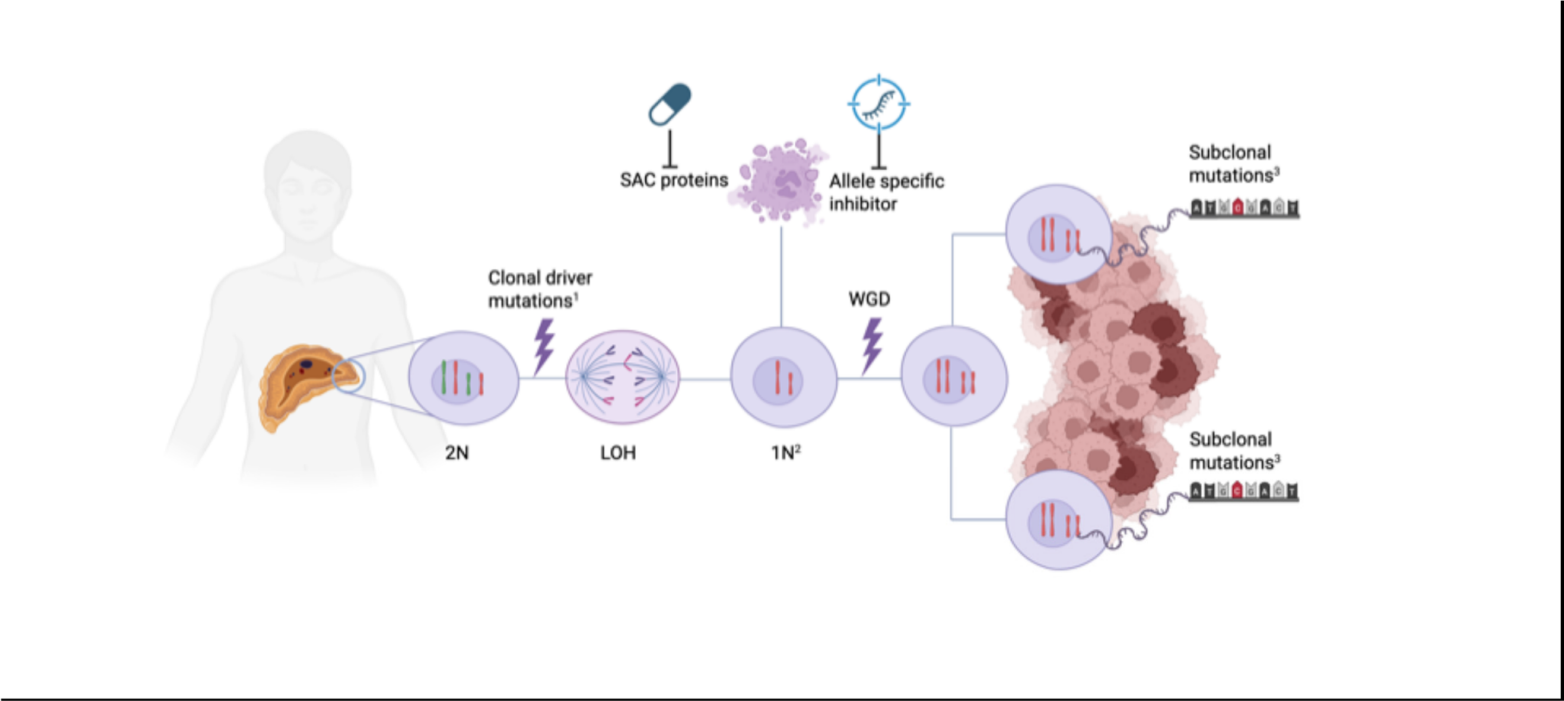
Proposed model of tumour evolution driven by loss of heterozygosity, whole-genome doubling, and subsequent subclonal diversification. A normal diploid (2 N) precursor cell acquires early clonal driver mutations¹ that promote tumour initiation. During mitosis, erroneous chromosome segregation leads to loss of heterozygosity (LOH), generating a haploid-like intermediate state (1 N). This unstable genome undergoes whole-genome doubling (WGD). As the tumour expands, additional subclonal mutations³ accumulate, contributing to intratumoral heterogeneity and the emergence of distinct subpopulations with variable fitness and treatment response. ¹ Early clonal driver mutations; ² Haploid-derived intermediate; ³ Later-arising subclonal mutations.

Our cohort of paired primary and relapse ACC tumour samples displayed evolutionary patterns dominated by large-scale chromosomal events. Widespread loss of heterozygosity followed by whole-genome doubling occurred truncal in tumorigenesis and preceded many point mutations, arguing against a stepwise adenoma-to-carcinoma progression (Fig. 6). Despite interpatient heterogeneity, tumours converge on a shared dependency on tolerance of aneuploidy, reflected in recurrent alterations affecting cell-cycle control, mitotic fidelity, and chromatin regulation. These findings highlight copy-number alterations as important determinants of ACC biology and prognosis, and point to aneuploidy-associated vulnerabilities as potential therapeutic targets.

## Supplementary Information

Below is the link to the electronic supplementary material.

Supplementary Material 1

Supplementary Material 2

Supplementary Material 3

## Data Availability

All data can be obtained from the corresponding author upon reasonable request. The data analyses presented in this paper were performed using publicly available software as detailed in the Methods section.
