# Supplementary material for: Genomic Catastrophe Defines the Evolutionary Trajectory of Adrenocortical Carcinoma
Source: Endocr Pathol. 2026 Jun 10;37(1):25. doi: 10.1007/s12022-026-09922-2 (PMC13249746; doi:10.1007/s12022-026-09922-2)

Genomic Catastrophe Defines the Evolutionary Trajectory of Adrenocortical Carcinoma

Samuel Backman[1], Fredrik Axling[1], Liang Zhang[2], Johan Botling[3], C Christofer Juhlin[4,5], Elham Barazeghi[1], Britt Skogseid[2], Branislav Klimácek[1], Matilda Annebäck[1], Jan Zedenius[6,7], Olov Norlén[1], Peter Stålberg[1], Joakim Crona[2], Tobias Åkerström[1]*

Affiliations:

[1]Department of Surgical Sciences, Uppsala University, Uppsala, Sweden.

[2]Department of Medical Sciences, Uppsala University, Uppsala, Sweden.

[3]Department of Laboratory Medicine, Institute of Biomedicine, University of Gothenburg, Gothenburg, Sweden.

[4]Department of Oncology - Pathology, Karolinska Institutet, Stockholm, Sweden.

[5]Department of Clinical Pathology and Cancer Diagnostics, Karolinska University Hospital, Stockholm, Sweden

[6]Department of Molecular Medicine and Surgery, Karolinska Institutet, Stockholm, Sweden

[7]Department of Breast, Endocrine Tumors and Sarcoma, Karolinska University Hospital, Stockholm, Sweden

*Corresponding author

Tobias Åkerström

Department of Surgical Sciences, Uppsala University hospital.

75185, Uppsala, Sweden.

Supplementary Figure 1. A) Flowchart illustrating patient and sample inclusion for the cohort. From the total number of patients initially identified, cases were excluded based on defined criteria specified in the figure. **B)** Clinical course and treatment timeline of patients included in the cohort. Black arrowheads denote baseline sampling and relapse events, while black crosses mark time of death. Time is shown in years or months as specified on each axis.

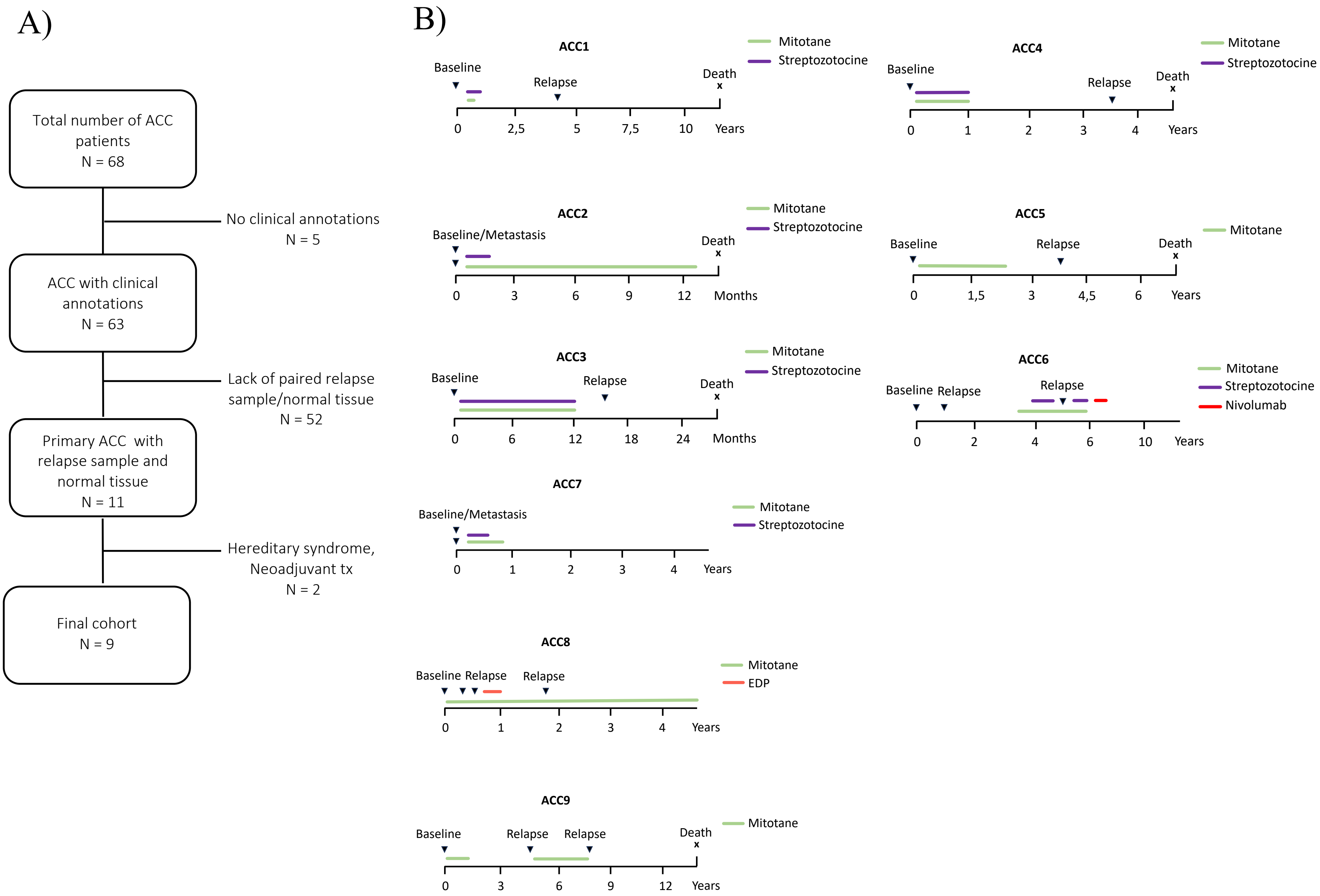

Supplementary Figure 2. Mutational signature analysis. A) Heatmap displaying different mutational signatures. B) Number of samples displaying the different mutational signatures.

A)

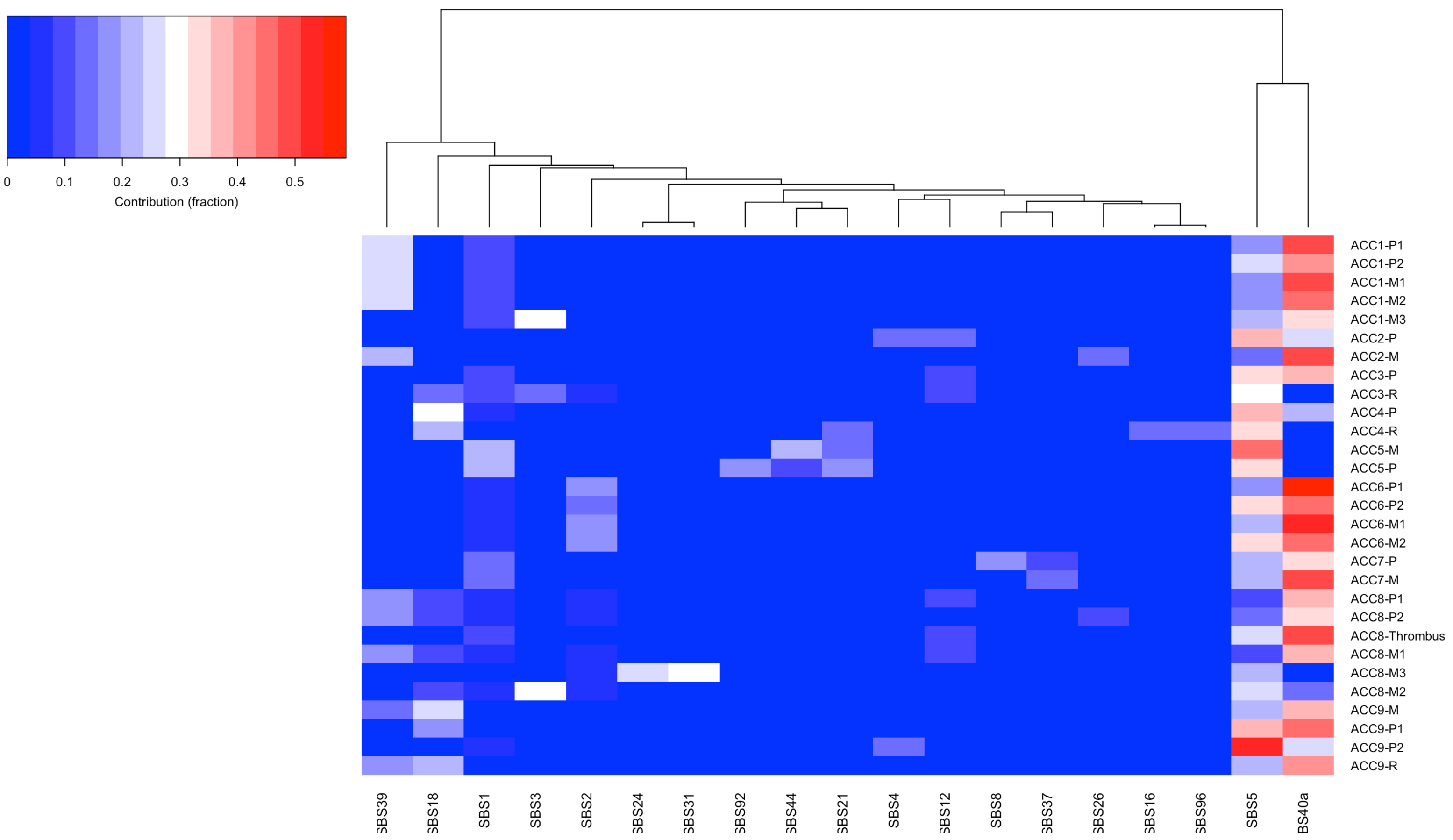

B)

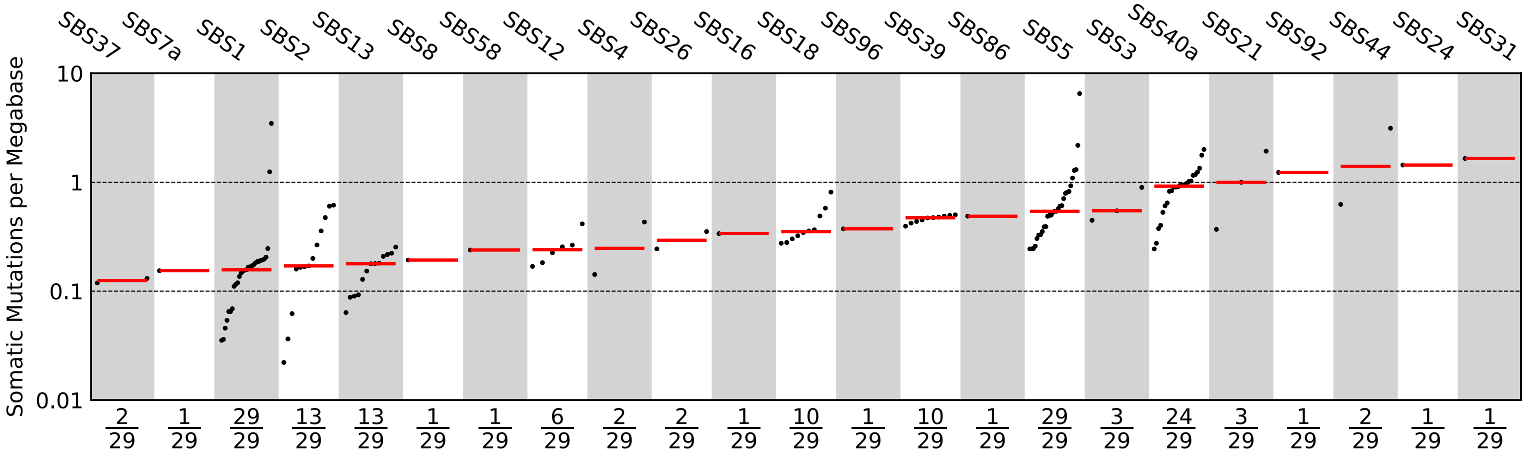

*Showing samples with counts more than 0

Supplementary Figure 3. **Genome-wide allele-specific copy number profiles in adrenocortical carcinoma (ACC).** Allele-specific copy number plots are shown for five representative ACC samples. Each panel displays the total copy number (brown) and the minor allele copy number (green) across all autosomes (x-axis). The y-axis indicates the absolute copy number.

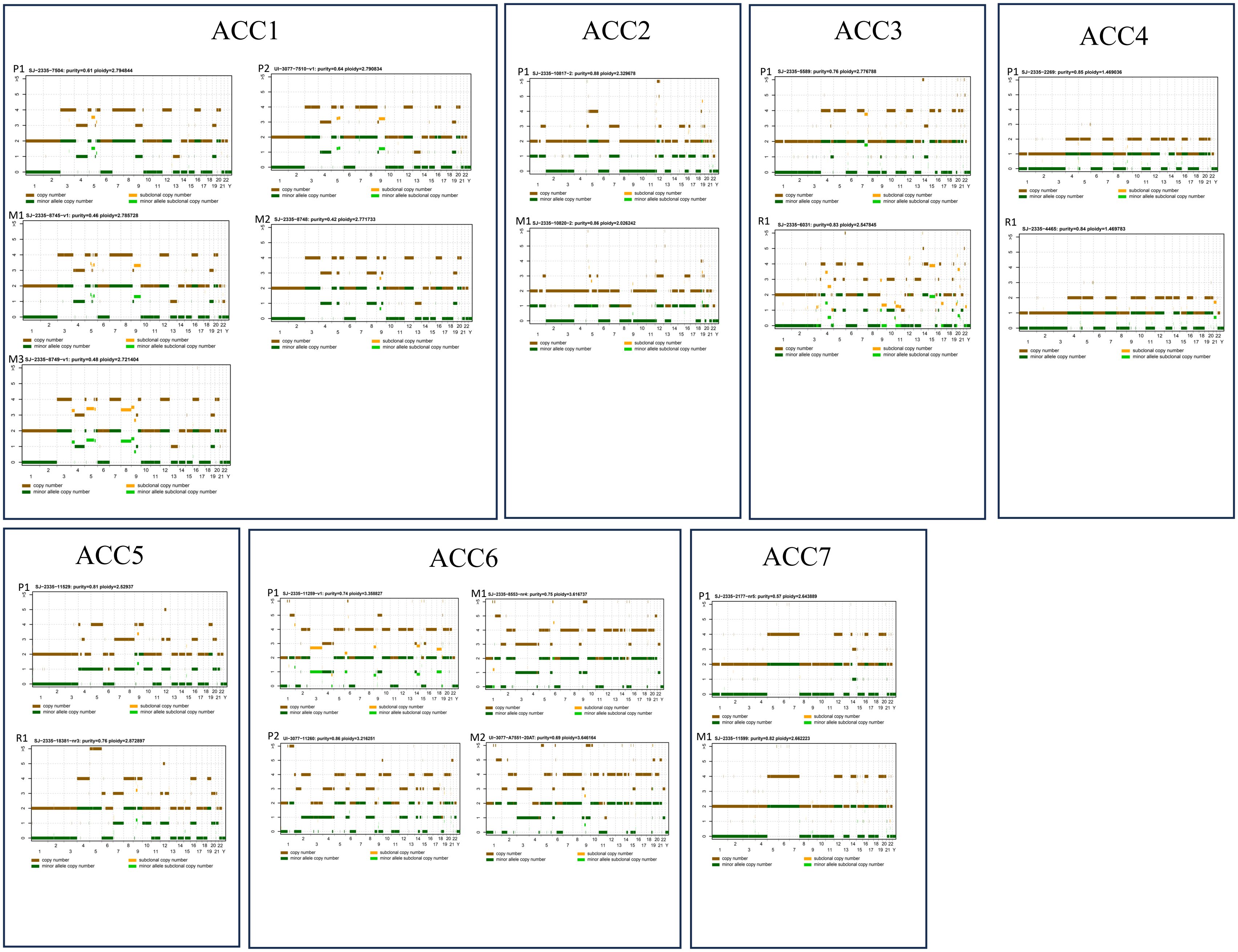

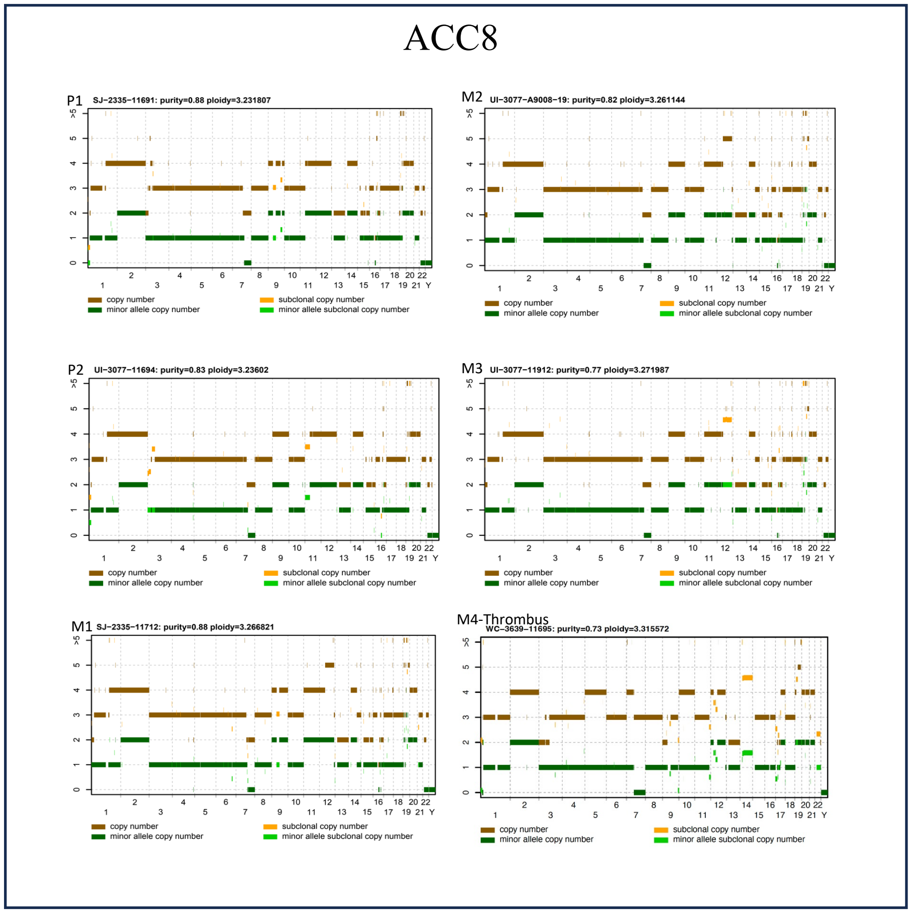
ACC8
P1
SJ-2335-11691: purity=0.88 ploidy=3.231807
M2
UI-3077-A9008-19: purity=0.82 ploidy=3.261144
P2
UI-3077-11694: purity=0.83 ploidy=3.23602
M3
UI-3077-11912: purity=0.77 ploidy=3.271987
M1
SJ-2335-11712: purity=0.88 ploidy=3.266821
M4-Thrombus
WC-3639-11695: purity=0.73 ploidy=3.315572
copy number
minor allele copy number
subclonal copy number
minor allele subclonal copy number

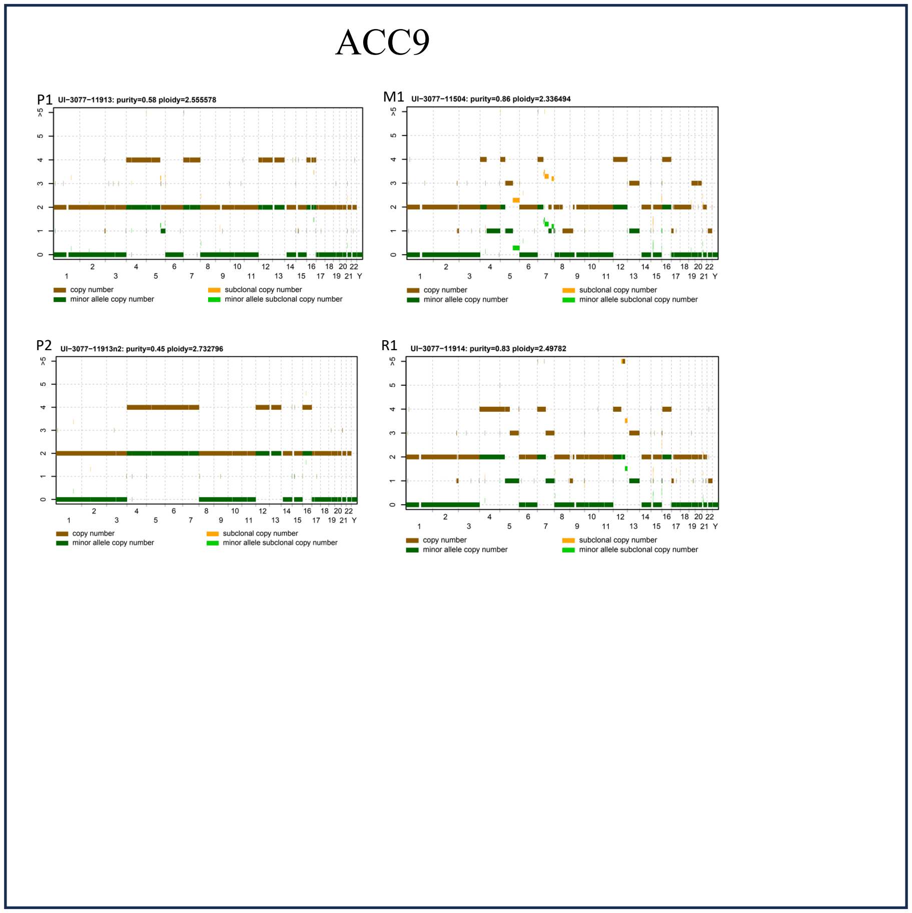
ACC9
P1
UI-3077-11913: purity=0.58 ploidy=2.555578
M1
UI-3077-11504: purity=0.86 ploidy=2.336494
P2
UI-3077-11913n2: purity=0.45 ploidy=2.732796
R1
UI-3077-11914: purity=0.83 ploidy=2.49782
copy number
minor allele copy number
subclonal copy number
minor allele subclonal copy number

Supplementary Figure 4. Association between Spindle assembly checkpoint proteins and overall survival in adrenocortical carcinoma (ACC) using the TCGA cohort. Kaplan–Meier survival analysis comparing overall survival between patients with high (red) and low (blue). Figures adapted using GEPIA.

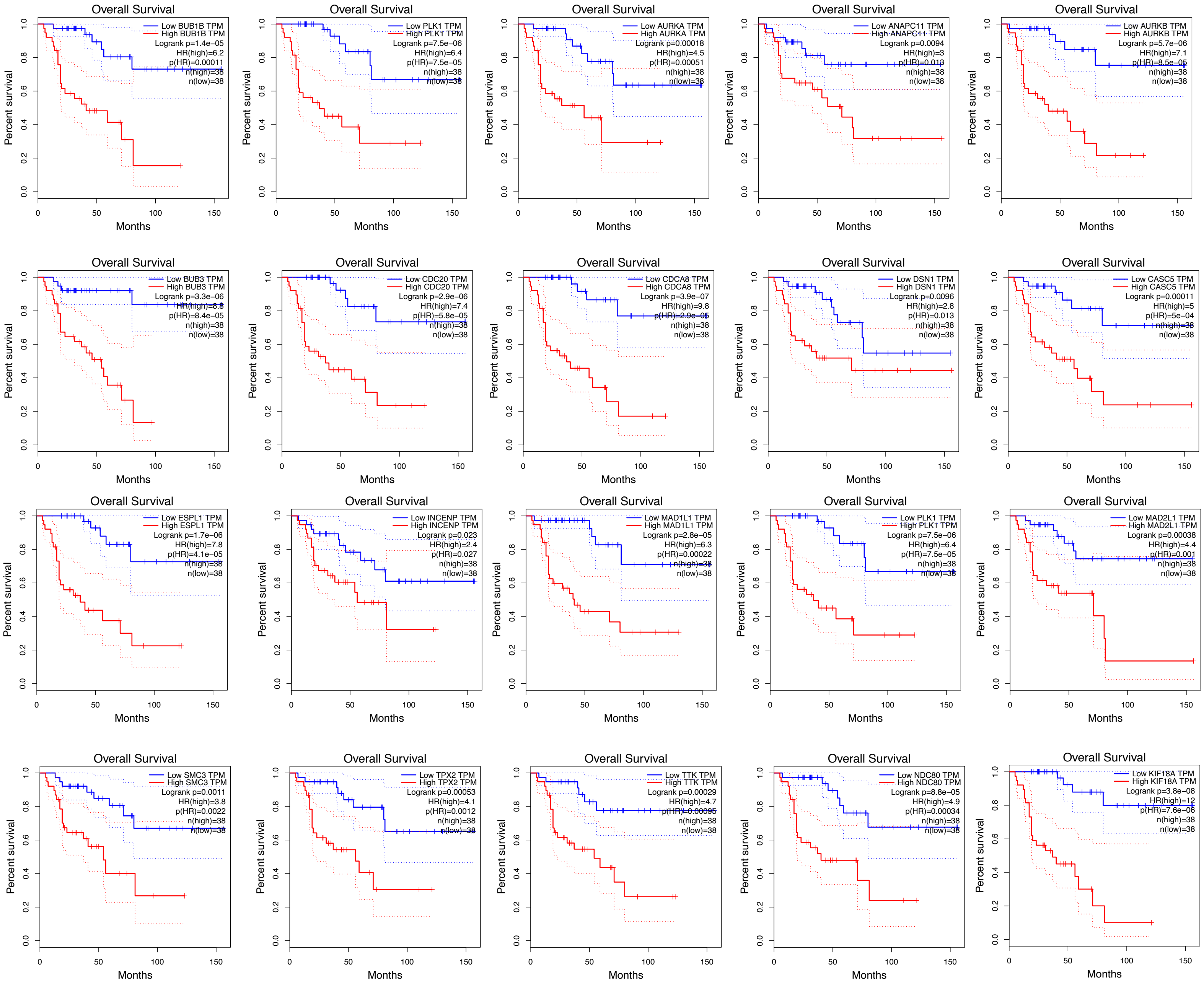

Supplementary Figure 5. Top row shows circos plots with structural rearrangements. Inner area depicts translocations; intrachromosomal deletions (blue) and duplications (red) are shown in the middle area. Middle row: Rainfall plots depicting mutation density. Bottom row: Rainfall plot of one tumour showing areas of hypermutation (Black square). P1: Primary tumour 1, R1: Relapse sample 1.

ACC1

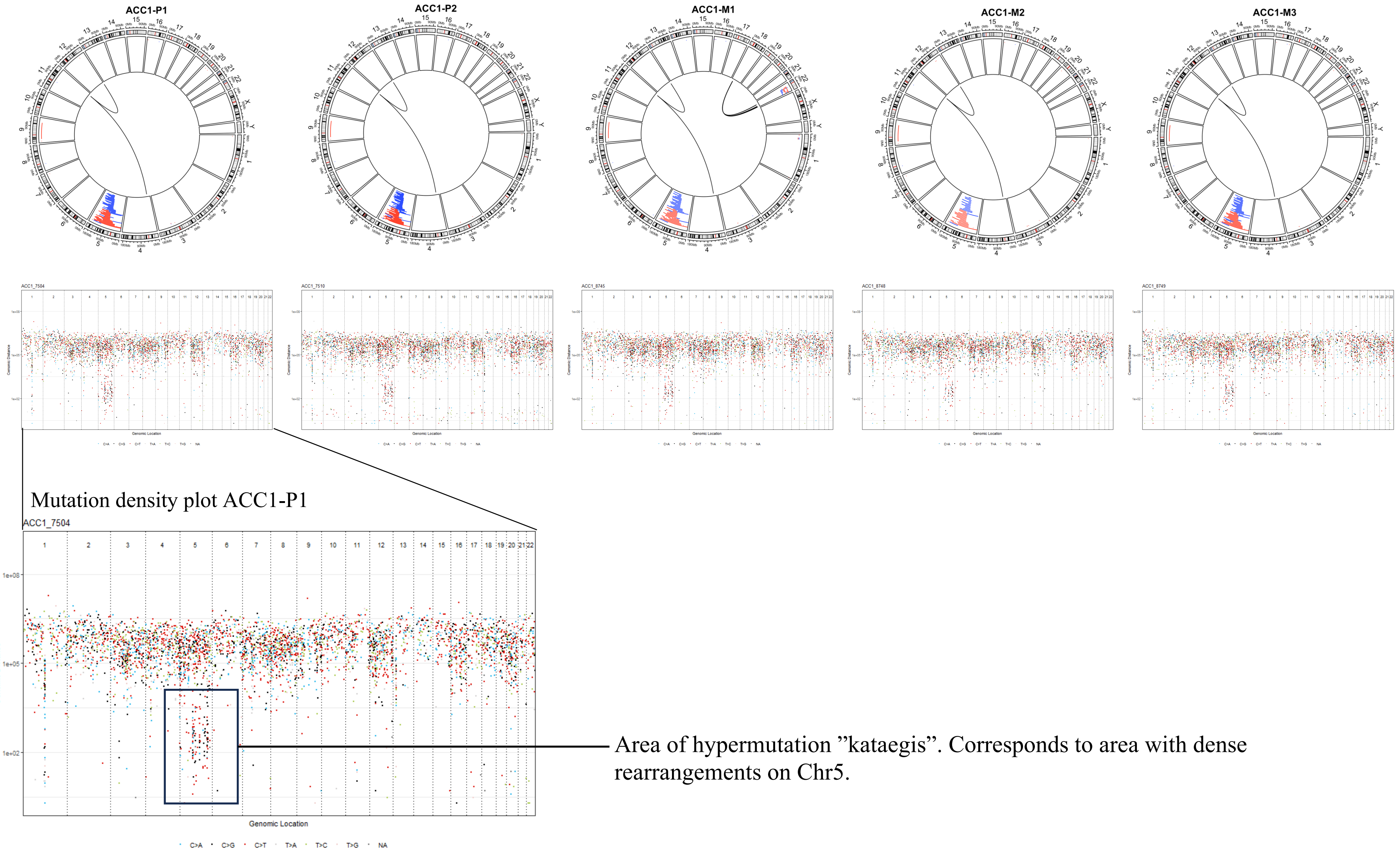

ACC2

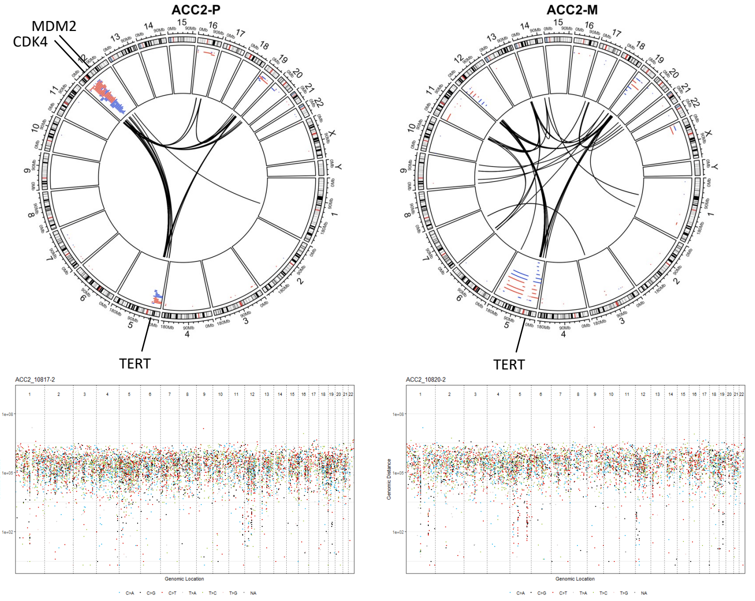

ACC3

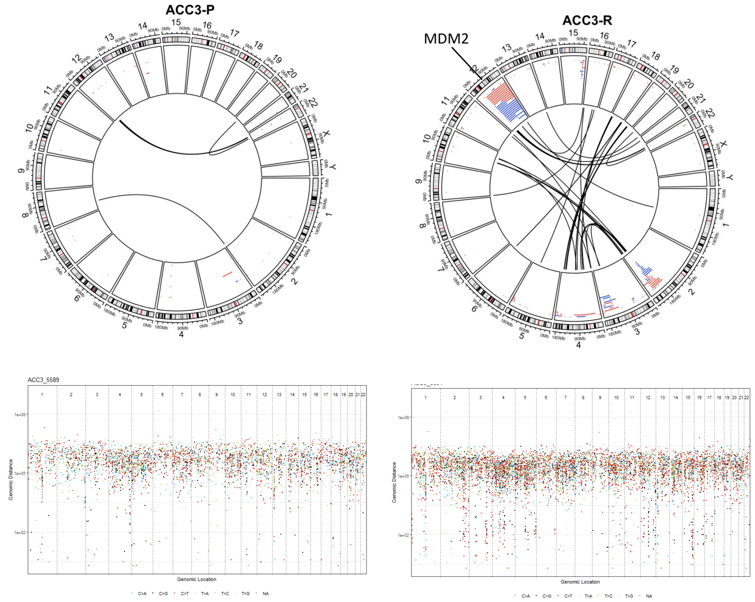

Mutation density plot ACC2_P1

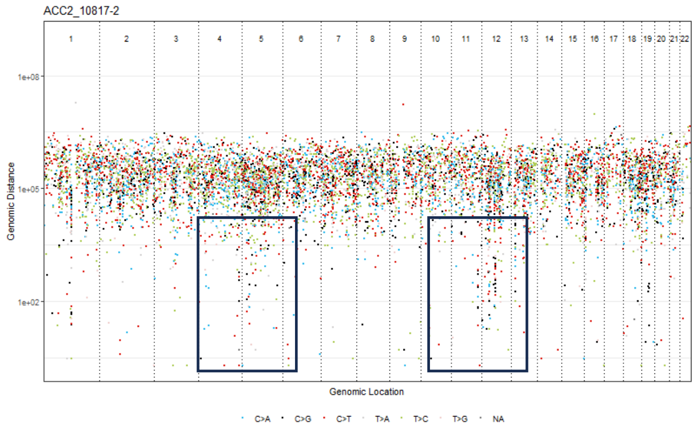

Mutation density plot ACC3_R1

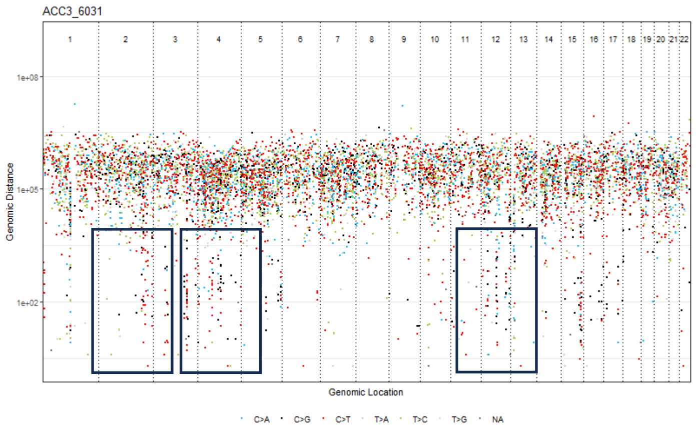

ACC4

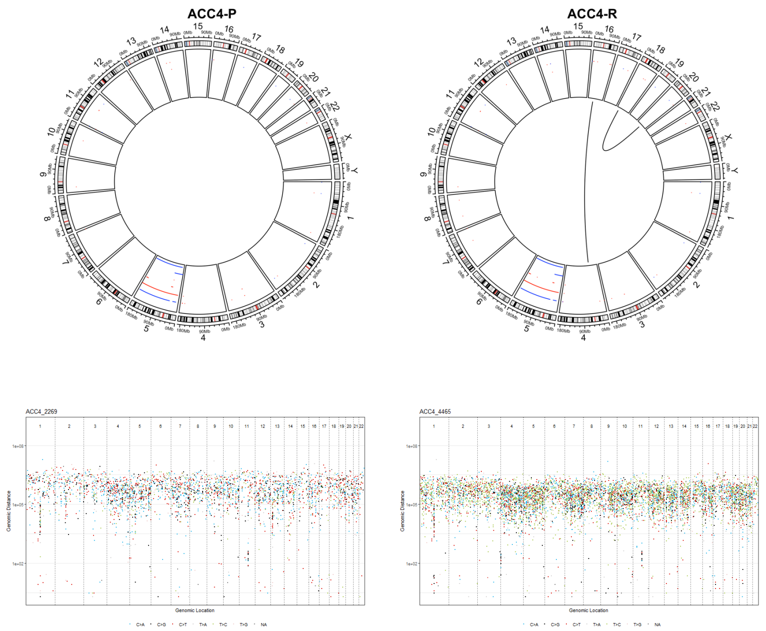
ACC4-P
ACC4-R
ACC4_2269
ACC4_4465
Genomic Location
Genomic Distance
C>A
C>G
C>T
T>A
T>C
T>G
NA

ACC5

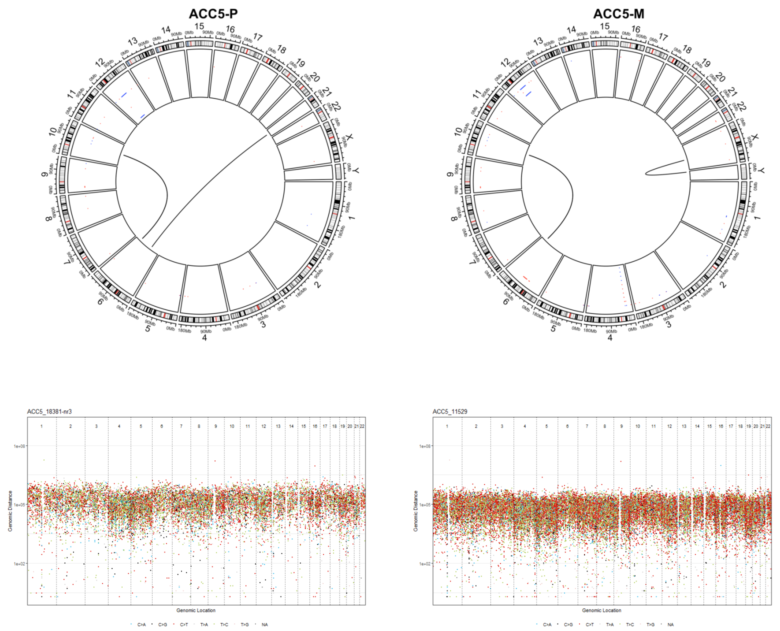
ACC5-P
ACC5-M
Genomic Location
Genomic Distance
C>A
C>G
C>T
T>A
T>C
T>G
NA

ACC6

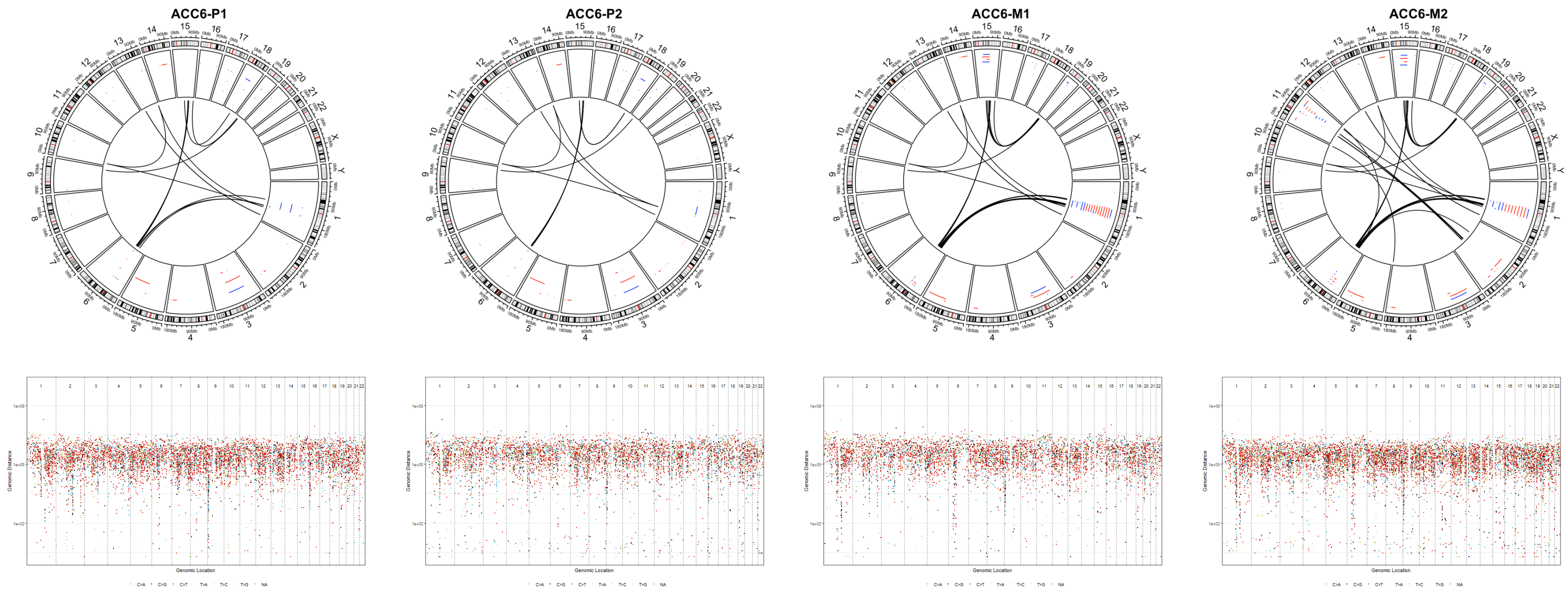

Mutation density plot ACC6_R2

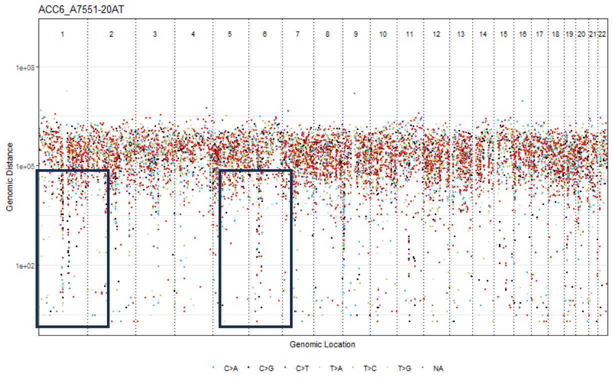

ACC7

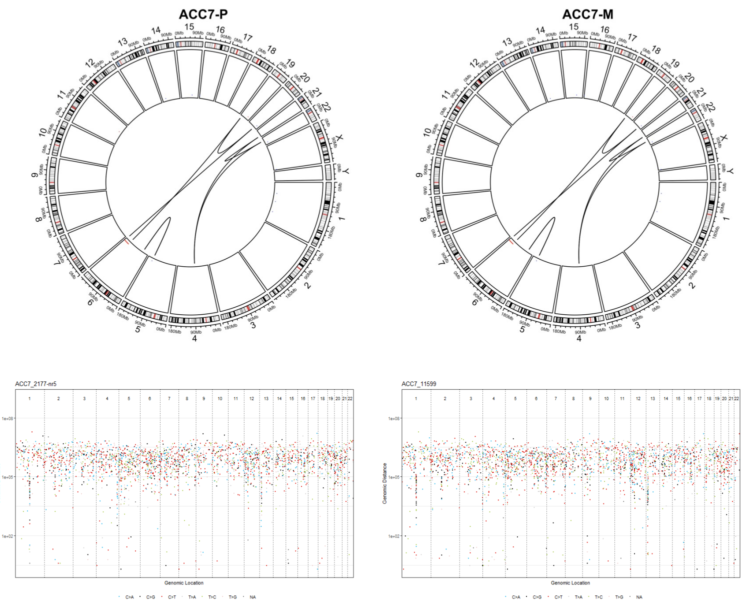
ACC7-P
ACC7-M

ACC8

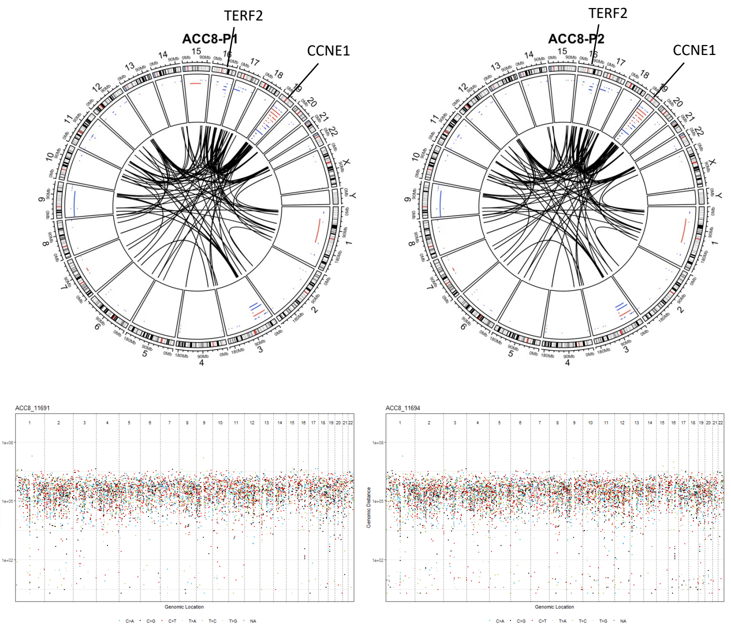
TERF2
ACC8-P1
CCNE1
TERF2
ACC8-P2
CCNE1

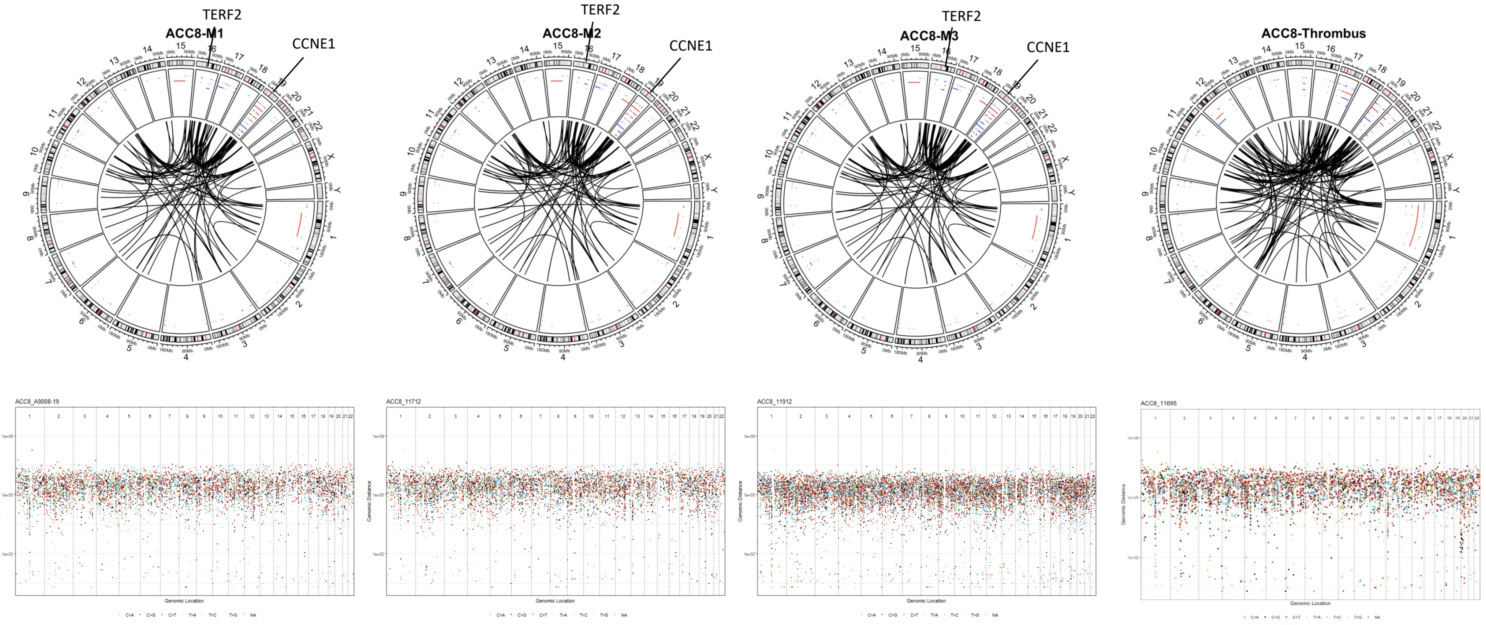
TERF2
ACC8-M1
CCNE1
TERF2
ACC8-M2
CCNE1
TERF2
ACC8-M3
CCNE1
ACC8-Thrombus

ACC9

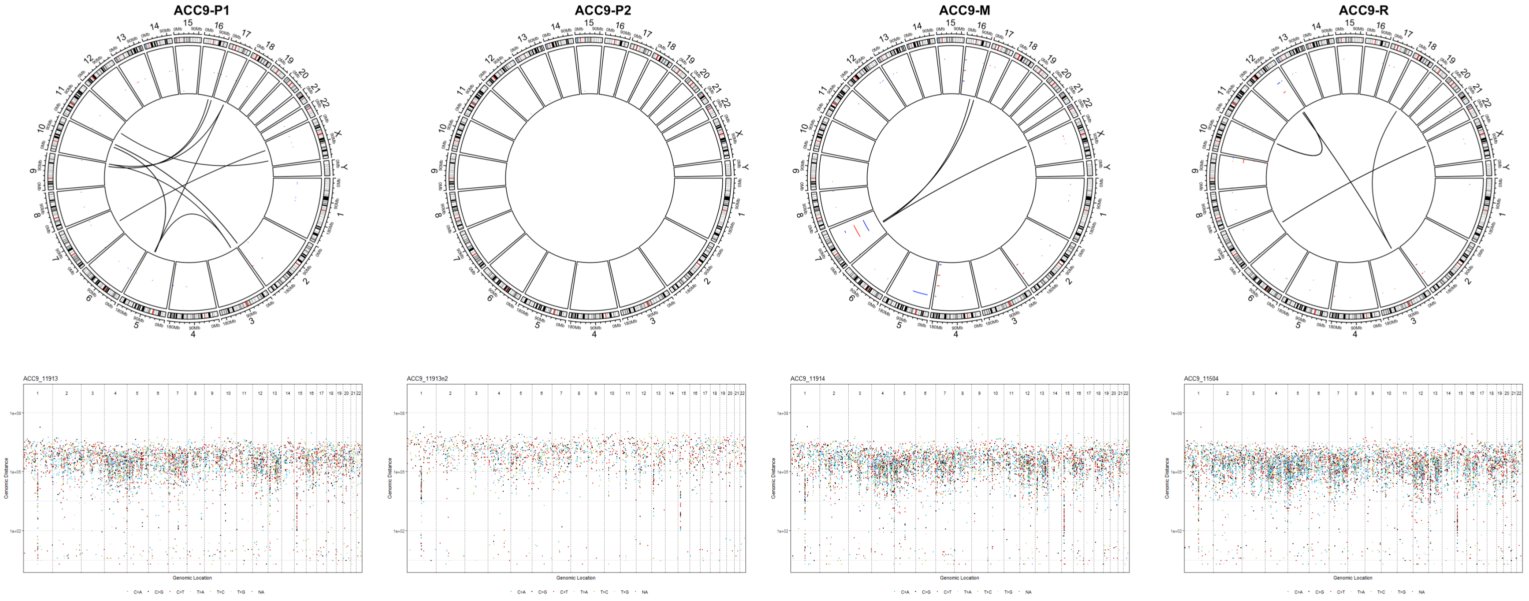

ACC9-P1
ACC9-P2
ACC9-M
ACC9-R
ACC9_11913
ACC9_11913m2
ACC9_11914
ACC9_11504
Genomic Distance
Genomic Location

Supplementary Figure 6. Clonal architecture inferred by PyClone analysis.
Scatter plot showing inferred cellular prevalence of somatic mutations based on PyClone clustering analysis. Each dot represents a somatic variant, plotted according to its cancer cell fraction (CCF) across samples. Colored circles indicate cellular populations inferred by Bayesian clustering, corresponding to distinct subclones within the tumour.

ACC6

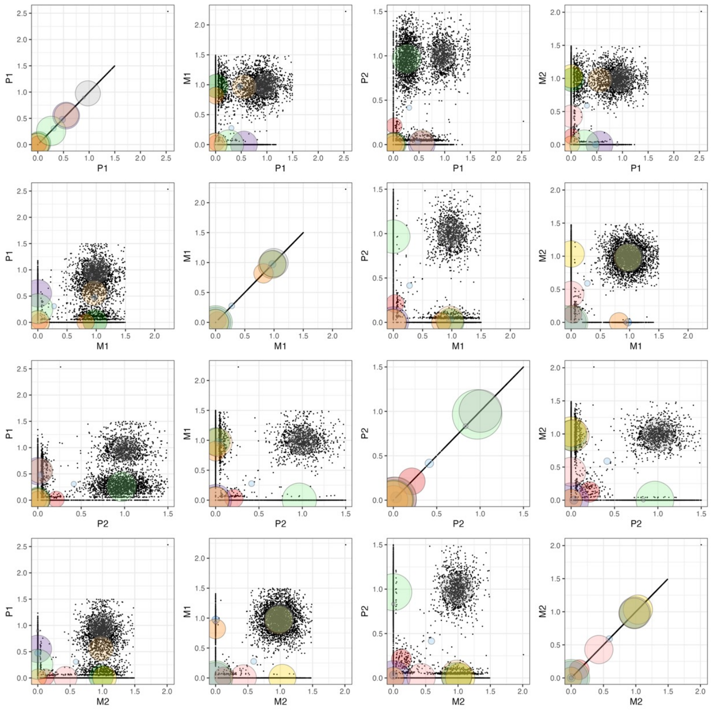

ACC8

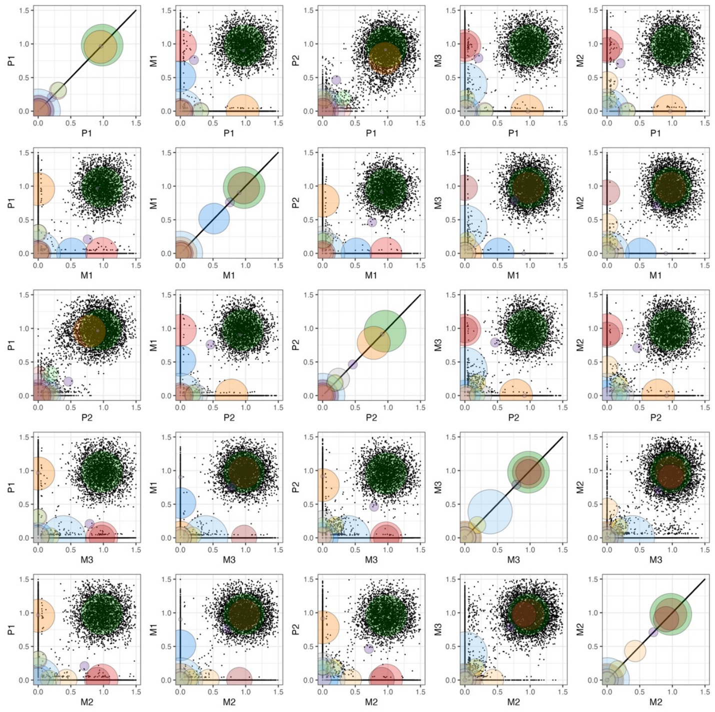

ACC9

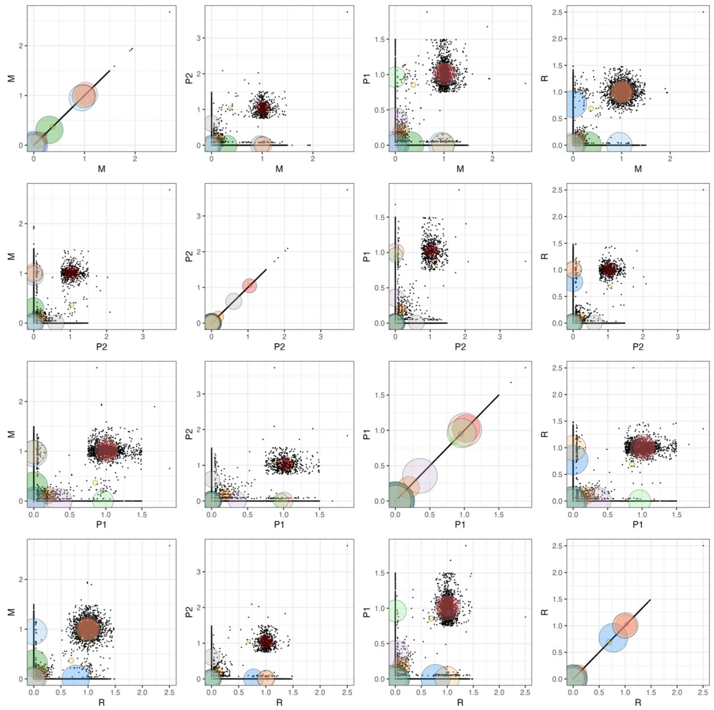

ACC1

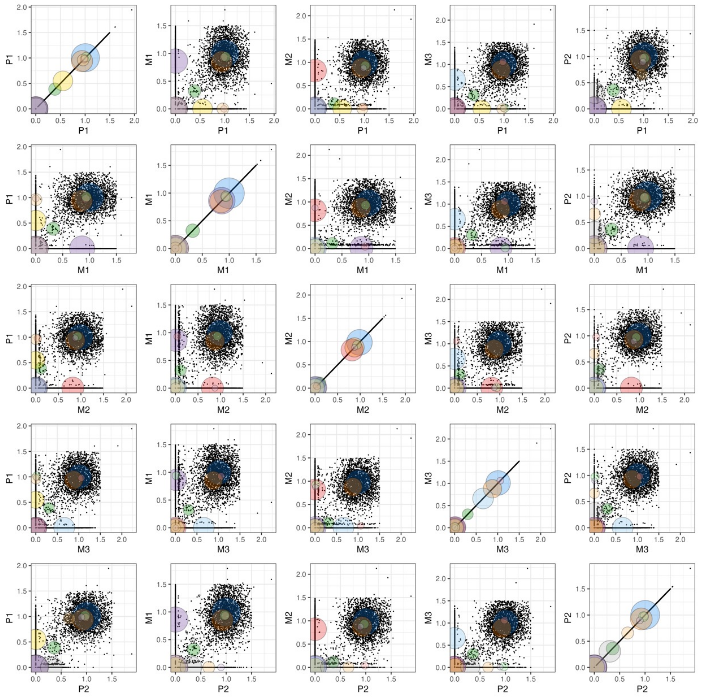

ACC2

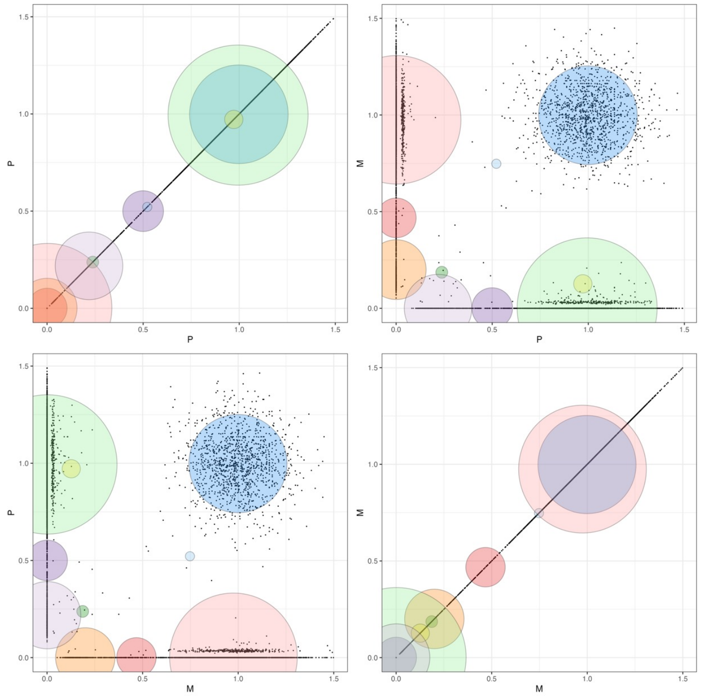

ACC3

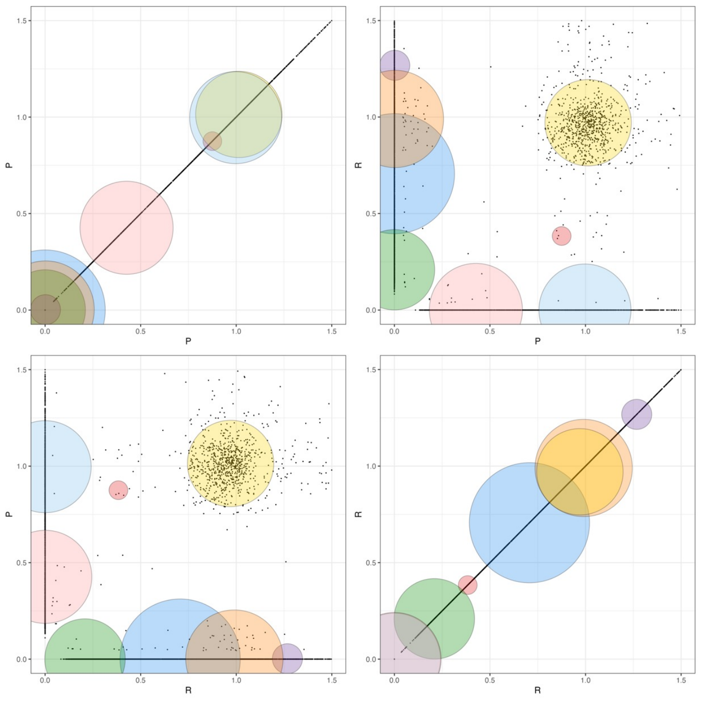

ACC4

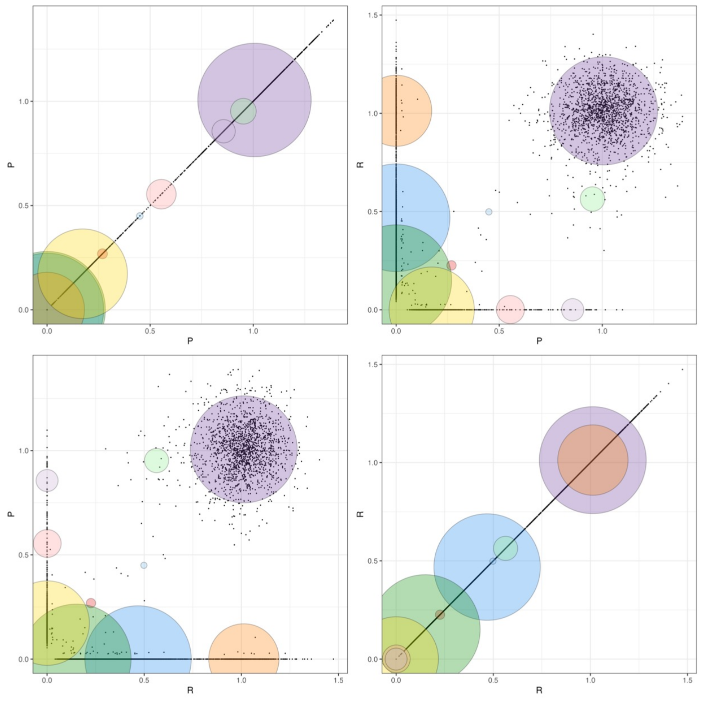

ACC5

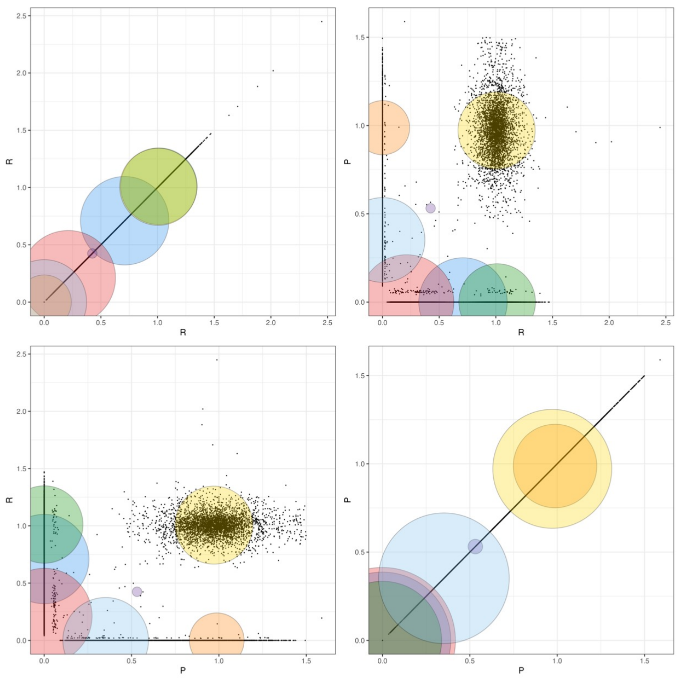

ACC7

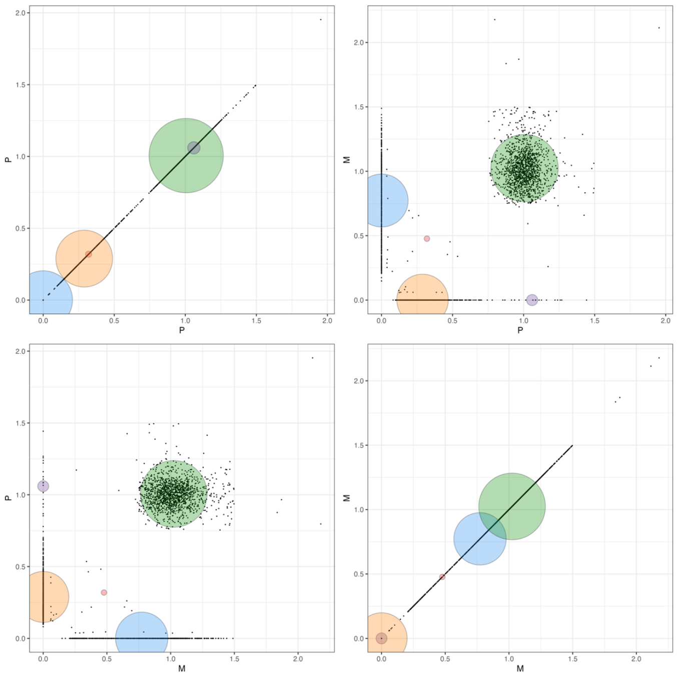

Supplementary Figure 7. Computer tomography of ACC6. A) CT scan of adrenals T=0 showing a 10x7 mm nodule (arrow) on the right adrenal measuring 33 Hounsfield units. B) CT scan at T+4 years with a 10 cm ACC.

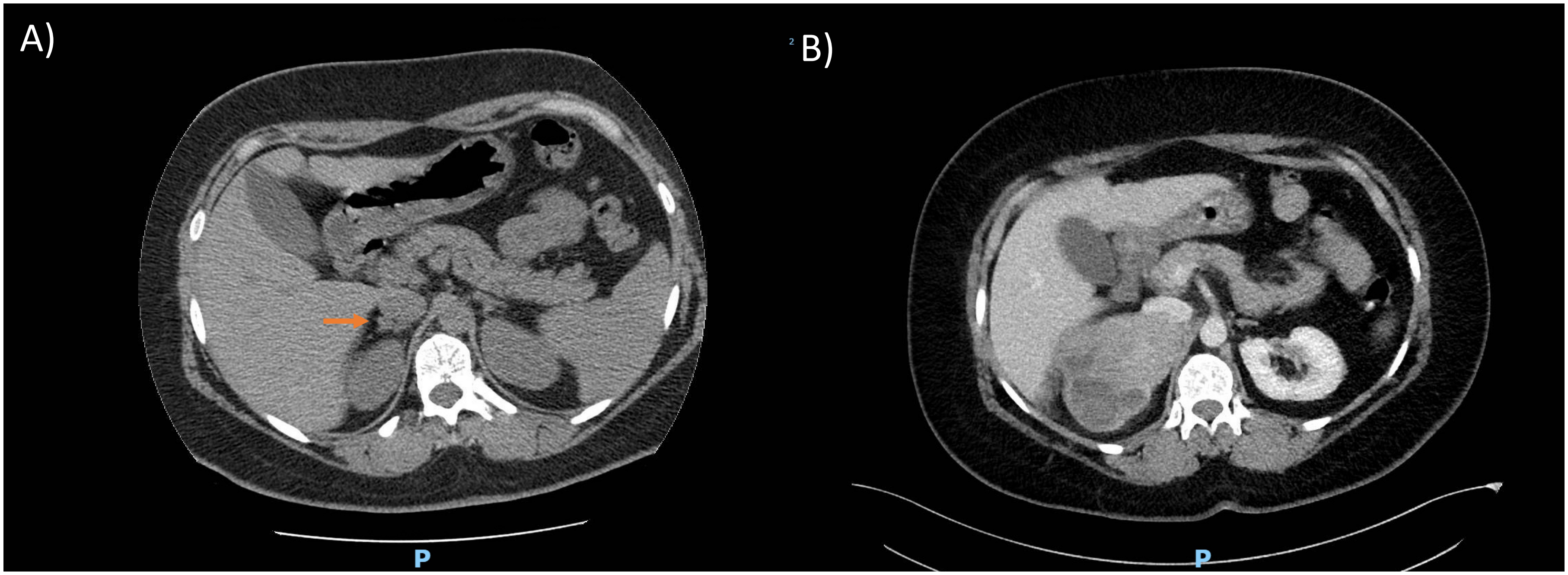

Supplementary Figure 8. A) Read frames for the c.1016-2A>G mutation in *ZNRF3*. Top row – ACC6_P1 with low allele frequency of *ZNRF3* mutation. Bottom row ACC6_P2. **B)** Visualization of the consequence on the mRNA molecule of the c.1016-2A>G mutation (Ensembl ENSG00000183579). C) Plot displaying genomic expression consequences of the c.1016-2A>G mutation in wild type ACC6_P1 and mutated ACC6_P2.

A)

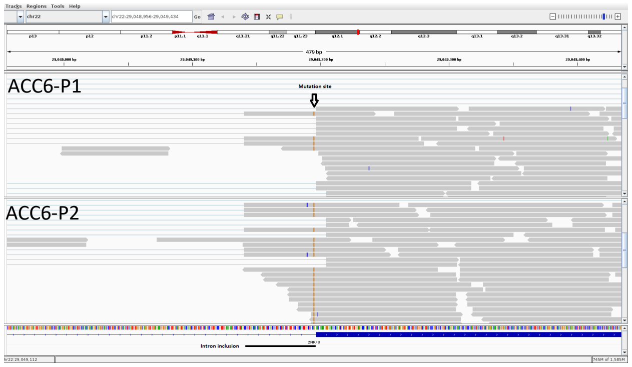

Inclusion of bases chr22:29,049,141-29,049,197

B)

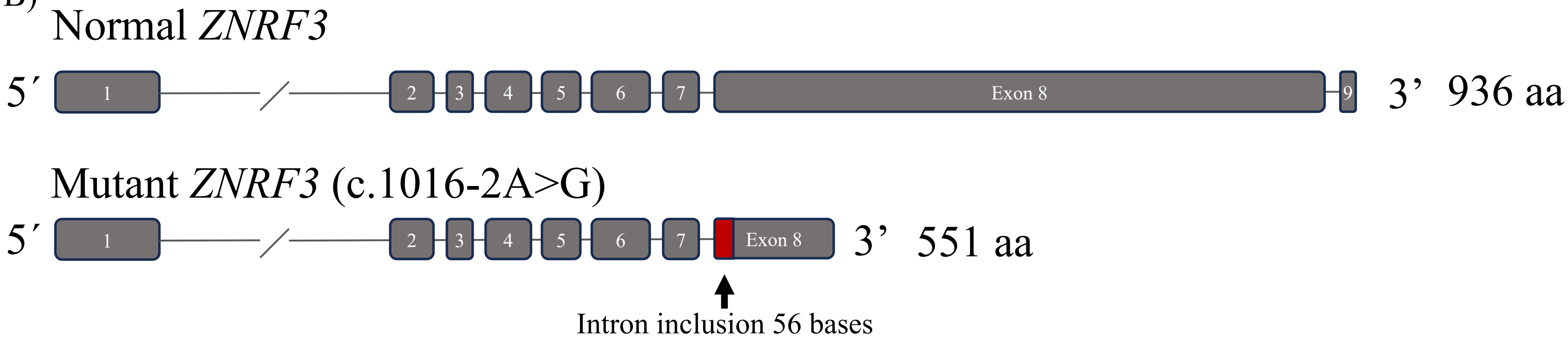

C)

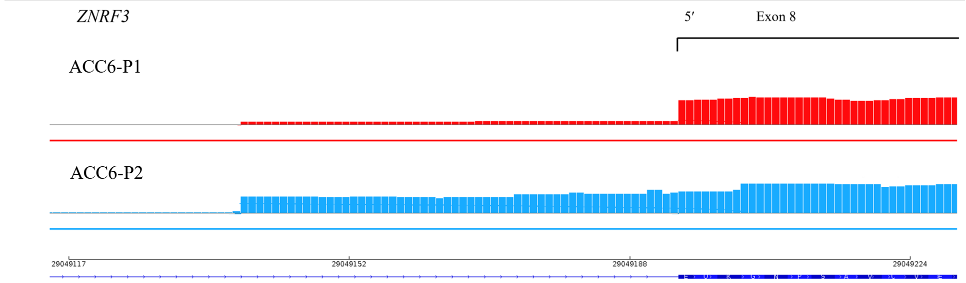

Supplementary Figure 8. Raw phylogenetic tree based on somatic single nucleotide substitutions identified across multiple tumours and matched germline DNA from patient ACC6, ACC8 and ACC9. Branch lengths represent the number of private or shared mutations between samples. .

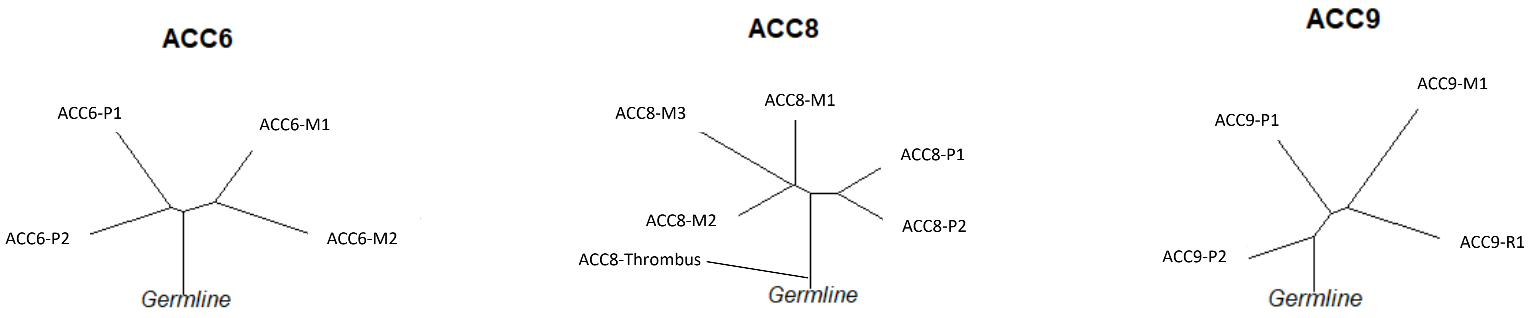

Supplementary Figure 9. A) Heatmap displaying pairwise correlation of RNA expression profiles among all tumour samples in the cohort. The x-axis denotes tumour type, and the y-axis lists individual samples. ACC6_11259_2_2; ACC8_11691_2 were used as controls. **B)** Gene ontology (GO) enrichment analysis. Functional enrichment analysis was performed using g:Profiler. The upper panel shows the distribution of enriched GO terms grouped by functional category. The lower panel lists the top enriched pathways ranked by adjusted *p*-value (Benjamini–Hochberg correction). **C)** Protein–protein interaction and pathway enrichment network of genes differentially expressed between primary and relapse samples. Network visualization of significantly enriched pathways derived from differentially expressed genes between primary and relapse ACC tumours. The analysis was performed using STRING. Interconnected clusters centered around genes involved in cell cycle regulation, mitotic spindle assembly, chromatin organization, and DNA repair.

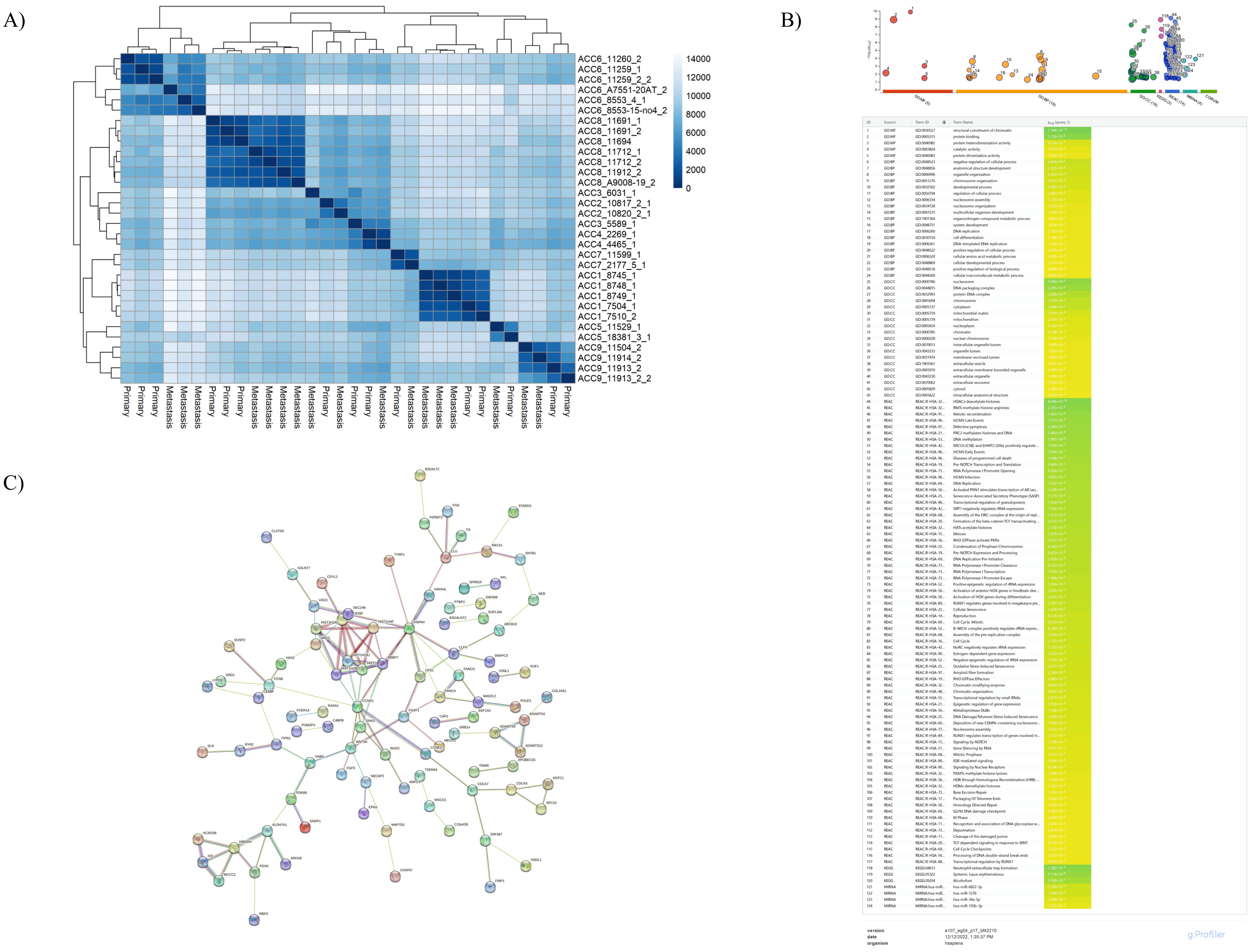

Supplementary Figure 10. Heatmap showing DNA methylation clustering. Each column represents an individual CpG site, and each row corresponds to a tumour sample. Color intensity reflects methylation level, ranging from low (blue) to high (yellow). Colored side bars indicate patient identity, white: primary; black: metastatic; grey: recurrent sample.

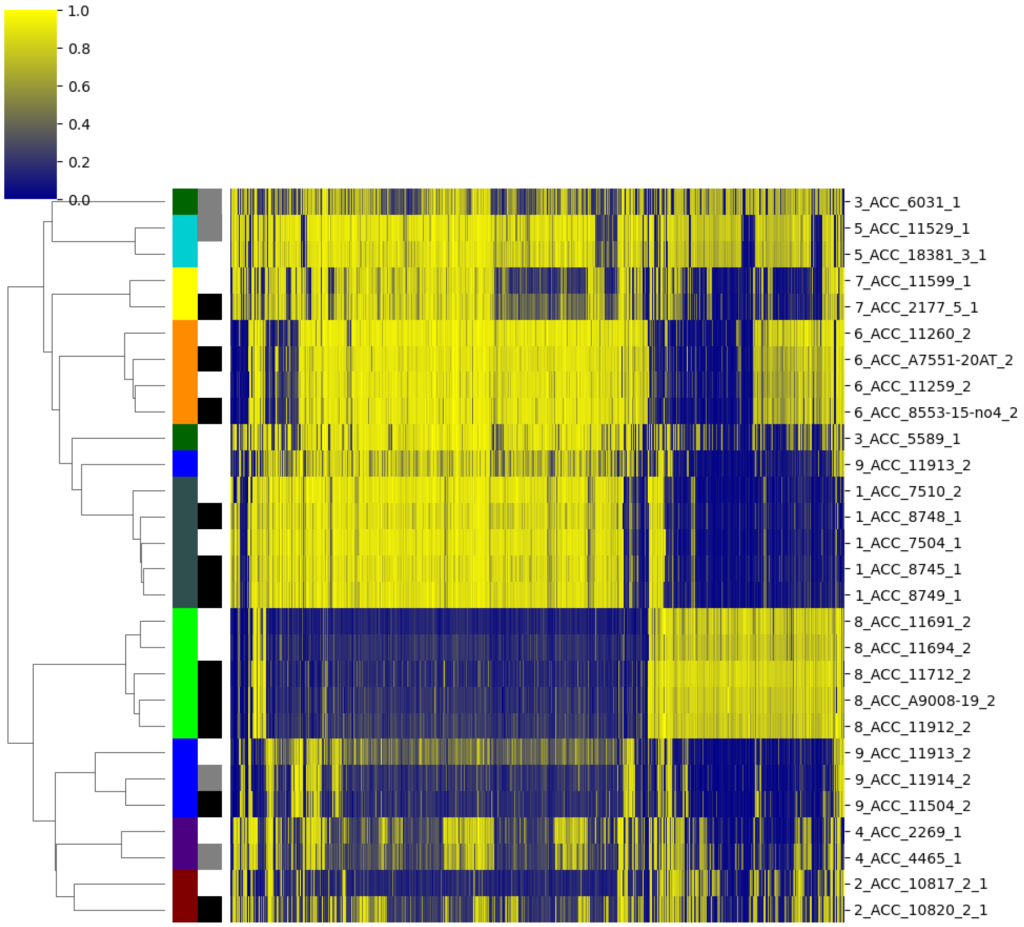

Supplement: Supplementary file 3 — Supplementary Material 3 [file 12022_2026_9922_MOESM3_ESM.pdf]
